# circMORC3-encoded novel protein negatively regulates antiviral immunity through synergizing with host gene MORC3

**DOI:** 10.1371/journal.ppat.1011894

**Published:** 2023-12-27

**Authors:** Linchao Wang, Weiwei Zheng, Xing Lv, Yanhong Song, Tianjun Xu

**Affiliations:** 1 Laboratory of Fish Molecular Immunology, College of Fisheries and Life Science, Shanghai Ocean University, Shanghai, China; 2 Laboratory of Marine Biology and Biotechnology, Qingdao National Laboratory for Marine Science and Technology, Qingdao, China; 3 Marine Biomedical Science and Technology Innovation Platform of Lin-gang Special Area, Shanghai, China; Stanford University, UNITED STATES

## Abstract

The protein-coding ability of circRNAs has recently been a hot topic, but the role of protein-coding circRNAs in antiviral innate immunity of teleost fish has rarely been reported. Here, we identified a novel circRNA, termed circMORC3, derived from Microrchidia 3 (MORC3) gene in *Miichthys miiuy*. circMORC3 can inhibit the expression of antiviral cytokines. In addition, circMORC3 encodes a novel peptide with a length of 84 amino acids termed MORC3-84aa. MORC3-84aa not only significantly inhibited TRIF-mediated activation of IRF3 and NF-κB signaling pathways, but also effectively suppressed the expression of antiviral cytokines triggered by RNA virus *Siniperca chuatsi rhabdovirus* (SCRV). We found that MORC3-84aa directly interacted with TRIF and negatively regulated TRIF protein expression. In addition, host gene MORC3 attenuates SCRV-induced IFN and ISG expression. Mechanistically, MORC3-84aa promotes autophagic degradation of TRIF by enhancing K6-linked ubiquitination and inhibits TRIF-mediated activation of the type I interferon signaling pathway. And the host gene MORC3 not only repressed IRF3 protein expression but also inhibited IRF3 phosphorylation levels. Our study shows that circMORC3 and host gene MORC3 played a synergistic role in viral immune escape.

## Author summary

Increasing evidence indicates that circRNAs have important functions, but the relationship between circRNAs encoded protein and TLR signaling pathway remains unknown, particularly in lower vertebrates. Here, we identified a circRNA-encoded protein that synergistically regulates the TLR signaling pathway with the host gene. Our results provided direct evidence that circMORC3 could negatively regulate TRIF by autophagic degradation through its encoded protein MORC3-84aa. In addition, we found that the host gene MORC3 can inhibit IRF3 protein expression and its phosphorylation level. Collectively, these findings provide important insights regarding the functional importance of circMORC3 and the host gene in viral immune escape.

## 1. Introduction

Innate immunity is the first line of defense against invading pathogens. This response of innate immunity is initiated by “pattern recognition receptors” (PRRs). In addition, invading pathogens are termed pathogen-associated molecular patterns (PAMPs) [1]. As important members of PRRs, Toll-like receptors (TLRs) are evolutionarily conserved receptors that play an indispensable role in PAMPs recognition. Type I IFN induction can be mediated by the nucleic acid-sensing toll-like receptor (TLR) family, and TLR activation triggers downstream signal adaptor proteins to regulate immune responses [2]. To successfully infect host cells, viruses have developed various strategies to antagonize the IFN response. When severe acute respiratory syndrome coronavirus 2 (SARS-CoV-2) infects humans, the viral ORF9b protein inhibits type I and type III IFN production induced by SeV and poly (I:C) and activation of the TLR3-TRIF signaling pathway [3]. Therefore, TIR domain-containing adaptor inducing interferon-β (TRIF) is an essential adaptor protein and plays a critical role in the innate immune response. As an important connector protein of the TLR signaling pathway, TRIF promotes TLR signaling and activates the transcription factors NF-κB and IRF3, leading to the production of pro-inflammatory cytokines and type I IFN [4,5]. To date, some negative regulators of TRIF have been identified and characterized. For example, TRIM8 mediates K6- and K33-associated TRIF polyubiquitination, therefore negatively regulating transcriptional induction of type I IFNs in primary murine cells [6]. Although the role of mammalian TRIF in antiviral immunity has been extensively studied, the mechanism of negative regulation of TRIF by non-coding RNAs in lower vertebrates has been less well studied; therefore, there is an urgent need to explore the mechanism of non-coding RNA regulation of TRIF-mediated signal transduction.

Recently, it has been found that circular RNAs (circRNAs) are involved in the development and progression of viral infections and host immune responses [7]. Circular RNAs are single-stranded covalently closed ncRNAs without 5’caps and 3’end poly(A) tails [8]. Studies have shown that circRNAs are important regulators of various pathologies and cell physiology, for example, circRNAs can act as miRNA sponges, control transcription, regulate protein localization and perform mRNA splicing [9–12]. In recent years, a growing number of studies have shown that some circRNAs can be translated into new proteins or isoforms with new physiological roles [13]. As a typical mechanism of circRNA initiation of translation, the internal ribosome entry site (IRES) element of circRNAs can directly recruit ribosomes to the internal site of the circRNA to initiate translation [14,15]. In addition to the above-metioned regulation functions of circRNAs, several studies have shown the host genes of circRNAs might be related to the mechanism of circRNA in the process of disease development. For example, circRNAs have also been shown to directly interact with the host gene in Osteoarthritis, which results in altered parent gene expression and activating autophagy [16]. However, the regulatory function of circRNAs with host genes has been less studied in innate immunity.

As a highly conserved nuclear protein superfamily member, MORC is found in humans, mice, nematodes, plants and mucilage [17]. The conserved character of MORC suggests that it is important in multicellular organisms [18]. Currently, MORCs are considered to be disease genes involved in various diseases and oncogenes for cancer progression and are expected to be important biomarkers for diagnosis and treatment [19]. Like other members of the same family, MORC protein has three typical domain structures: a conserved GHKL-ATPase domain at N-terminus, a conserved CW-type zinc finger domain in the middle and several coiled-coil domains at C-terminal. More importantly, these domains are responsible for the roles of MORC in gene regulation, protein-protein interaction and DNA damage repair [20–22]. MORC3 has been reported to be associated with certain immune diseases [23,24]. For example, influenza virus infection leads to increased expression of NXP2/MORC3, and silencing NXP2/MORC3 leads to a decrease in viral mRNA [25]. However, whether circRNA derived from the parental gene MORC3 can perform a role in antiviral immunity is unknown.

Here, we not only characterized the inhibitory effect of MORC3 on IFN activation, but also identified a circRNA derived from the host gene MORC3 that encodes a novel 84-amino-acid protein, termed as MORC3-84aa. MORC3-84aa negatively regulates TRIF-mediated antiviral innate immune response, revealing the functional importance of circMORC3 and the host gene in innate immunity.

## 2. Results

### 2.1 Characterization of circMORC3 involved in host innate immunity

**Fig 1:**
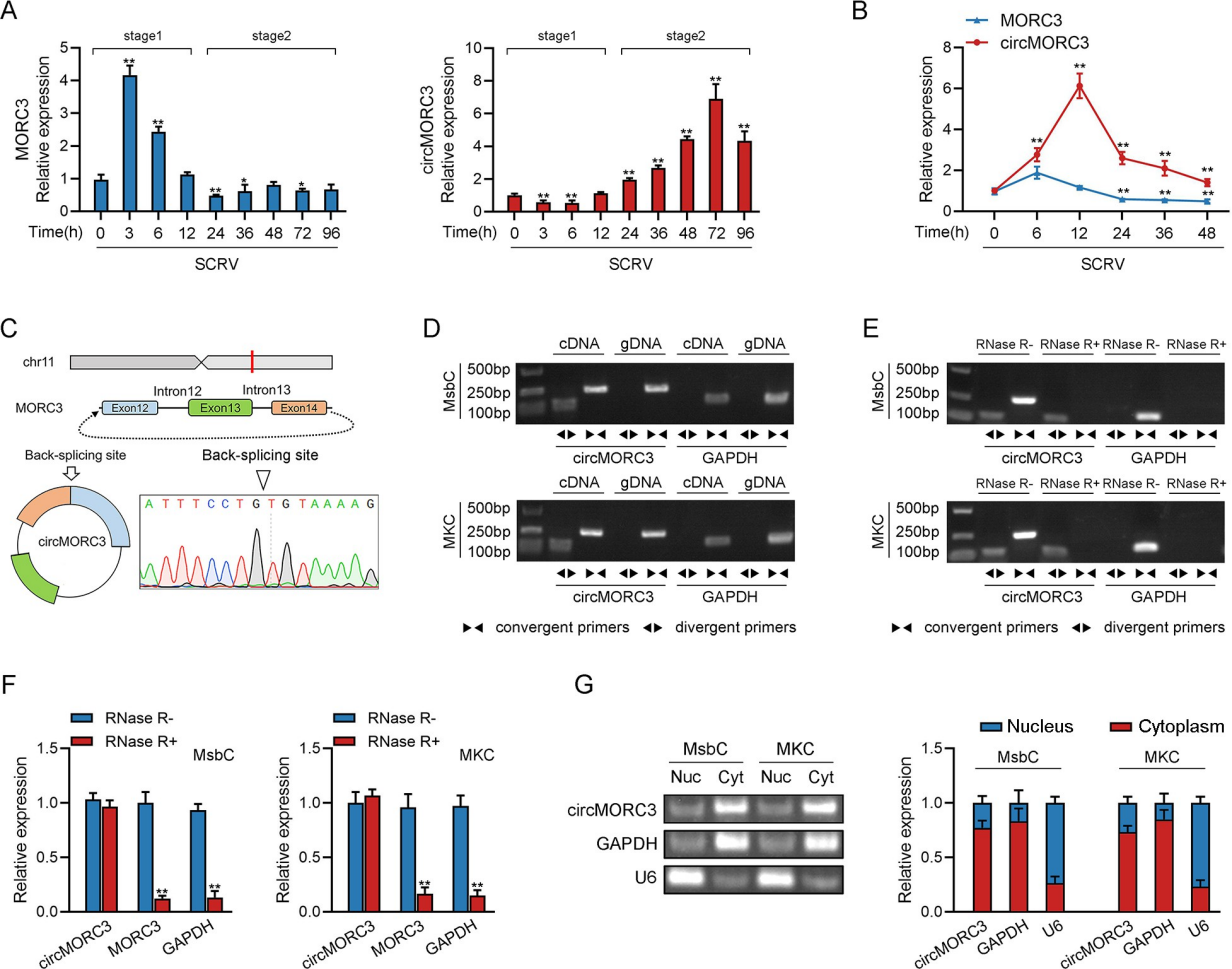
Expression profiles and characterization of circMORC3. (A) The expression levels of circMORC3 and MORC3 in liver samples after SCRV infection were detected by qRT-PCR. (B) mRNA levels of circMORC3 and linear MORC3 in MsbC cells measured by qRT-PCR after SCRV infection. (C) Confirmed the head-to-tail splicing of circMORC3 in the circMORC3 PCR product by Sanger sequencing. (D) PCR detection of circMORC3 and linear transcript of MORC3 by divergent and convergent primers in cDNA and gDNA of MsbC and MKC cells. (E) The stability of circMORC3 and MORC3 mRNA was detected by RT-PCR assay in the presence or absence of RNase R. (F) The stability of circMORC3 and MORC3 mRNA in MsbC and MKC cells was detected by qRT-PCR assay in the presence or absence of RNase R. (G) The existence of circMORC3 in MKC and MsbC was analyzed by nucleic acid electrophoresis and qPCR. All data presented as the means ± SE from at least three independent triplicated experiments. **, *p* < 0.01; *, *p* < 0.05 versus the controls.

To screen for immune-related candidate circRNAs, we compared the expression levels of circRNA after SCRV infection using RNA-seq data (GenBank accession no. PRJNA685924) and found that the expression of circMORC3 after SCRV infection was significantly upregulated. We further treated *M*. *miiuy* with SCRV, and sampled tissue at different times to extract RNA. Considering that circRNA is generated by linear RNA splicing, expression levels of host gene MORC3 and circMORC3 were also detected. Concerning the whole stress process of SCRV infection, it could be divided into two distinct stages: stage 1 and stage 2 (Figs 1A and S1A). In the first stage, the expression of MORC3 was significantly upregulated, whereas the expression of circMORC3 was not significantly changed. In the second stage, the expression of MORC3 returned to normal levels, whereas the expression of circMORC3 was significantly upregulated. However, SCRV-treated *M*. *miiuy* swim bladder cells (MsbC) further confirmed that both circMORC3 and MORC3 were upregulated, but the upregulation of circMORC3 was more significant compared to MORC3 under SCRV stimulation (Figs 1B and S1B).

Transcriptome sequencing showed circMORC3 to be 424 nt long. Blast analysis of the MORC3 gene using the *M*. *miiuy* whole genome library found that the MORC3 gene is located on chromosome 11 and is composed of 19 exons, and circMORC3 which is self-cyclized by exon12-exon14 and intron12-intron13 (Fig 1C). To confirm the objective existence of circMORC3, firstly, we designed the divergent primers of circMORC3 for RT-PCR amplification, and the amplification products were sequenced by Sanger, and it was confirmed that circMORC3 was spliced from beginning to end (Fig 1C). We then amplify the MORC3 gene using convergent primers and divergent primers to amplify circMORC3. cDNA and gDNA were extracted from *M*. *miiuy* kidney cells (MKC) and MsbC cells, respectively, and RT-PCR and agarose gel electrophoresis assays were performed. The results showed that circMORC3 was amplified from cDNA using only divergent primers, while no amplification products were observed from gDNA (Fig 1D).

Considering that stability is an important characteristic of circRNA, we used RNase R to confirm the stability of circMORC3. Results from RT-PCR and agarose gel electrophoresis analysis showed that circMORC3, rather than linear MORC3 or GAPDH, resisted digestion of RNase R (Fig 1E and 1F). After enrichment with RNase R enzyme, the relative expression level of circMORC3 did not increase, which may be due to the very low background level of circMORC3. Even after enrichment, the relative expression level of circMORC3 did not significantly increase. In addition, we investigated the distribution of circMORC3 by cytoplasmic nucleus isolation experiments and found that circMORC3 is mainly localized in the cytoplasm (Fig 1G). Therefore, these results indicate that circMORC3 is a stable circRNA and has potential functions in the innate immune response.

### 2.2 circMORC3 encoded a novel 84aa peptide termed MORC3-84aa

**Fig 2:**
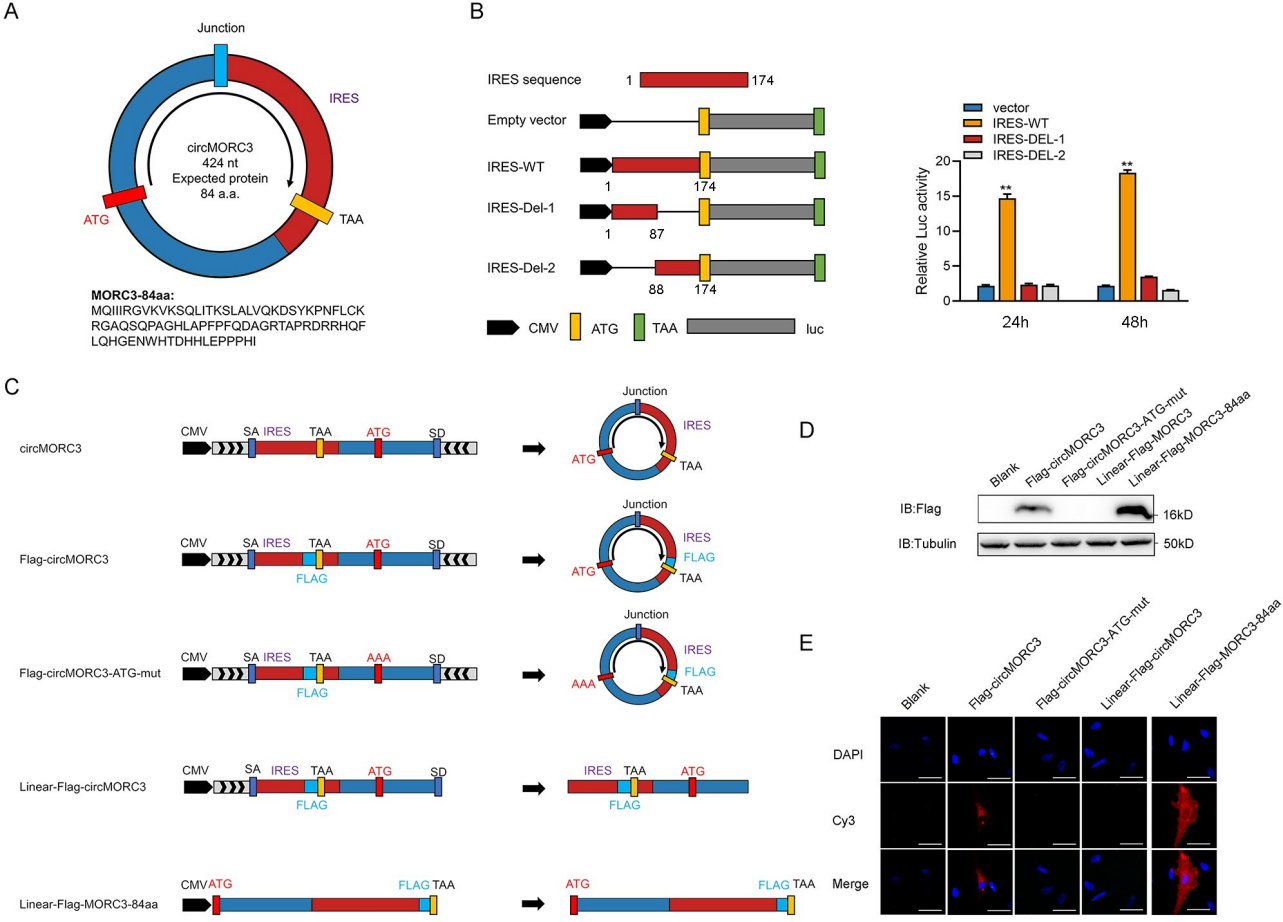
circMORC3 encodes a 84 amino acid novel protein, MORC3-84aa. (A) The predicted putative open reading frame (ORF) in circMORC3. (B) The putative IRES activity in circMORC3 was tested. (C) Plasmid construction pattern diagram of circMORC3. (D) Flag tag antibody was used to detect MORC3-84aa expression in HEK293 cells transfected with the above vectors. (E) The subcellular distribution of MORC3-84aa was analyzed by immunofluorescence in MsbC cells. Scale bars = 10 μm. All data presented as the means ± SE from at least three independent triplicated experiments. **, *p* < 0.01; *, *p* < 0.05 versus the controls.

There is growing evidence that circRNA can serve as a template for protein. Therefore, we use the cORF pipeline script to search for circMORC3 assumed open reading frames (ORFs) [26]. We predict that the ORF of circMORC3 may encode 84 aa protein. ORF analysis showed that the reading frame of circMORC3 spanned the splicing site (Fig 2A). Computational analysis was used to identify the stem-loop like structure of precursor in circMORC3 (S1C Fig). IRESite was used to identify the IRES sequence of circRNA [27]. Bioinformatics analysis identified a putative internal ribosome entry site (IRES) sequence (174nt from ORF) of circMORC3 that is required in the translation initiation of 5’ cap-independent coding RNA. The full-length or truncated IRES sequences of circMORC3 were then cloned into the pGL3-Basic vector along with the CMV sequence, and the predicted IRES activity was detected using a dual-luciferase assay (left panel of Fig 2B), with the phRL-TK vector expressing the RLuc (*Renilla* luciferase) reporter gene serving as a negative control. The results showed that the assumed IRES sequence had a strong ability to initiate protein translation at different time points. However, truncated vectors did not show significant effects (right panel of Fig 2B). In addition, we established a set of vectors to verify the translatability of circMORC3. As shown in Fig 2C, the connection of endogenous circMORC3 is inside the ORF. A novel circMORC3 vector containing the Flag sequence was constructed to verify its protein-coding ability (Flag-circMORC3). For negative controls, the downstream flanking sequences were deleted (Linear-Flag-circMORC3). We then constructed a mutant circMORC3 overexpressing vector that lacks the protein-coding ability (Flag-circMORC3-ATG-mut). In addition, a linearized ORF plus Flag tag was cloned into a linear vector (Linear-Flag-MORC3-84aa). In addition, these overexpressing plasmids were validated through Sanger sequencing and qRT-PCR (S2A and S2B Fig). Western blot results showed that both Flag-circMORC3 and Linear-Flag-MORC3-84aa were able to produce MORC3-84aa protein in cells. However, this effect was lost in the Linear-Flag-circMORC3 and Flag-circMORC3-ATG-mut groups (Fig 2D). The distribution of MORC3-84aa in cells was further determined using the Flag antibody. Immunofluorescence results show that MORC3-84aa mainly distributed in the cytoplasm (Fig 2E). Taken together, our results proved that circMORC3 was translated into MORC3-84aa in an IRES-dependent manner.

### 2.3 circMORC3 inhibits host innate immunity

**Fig 3:**
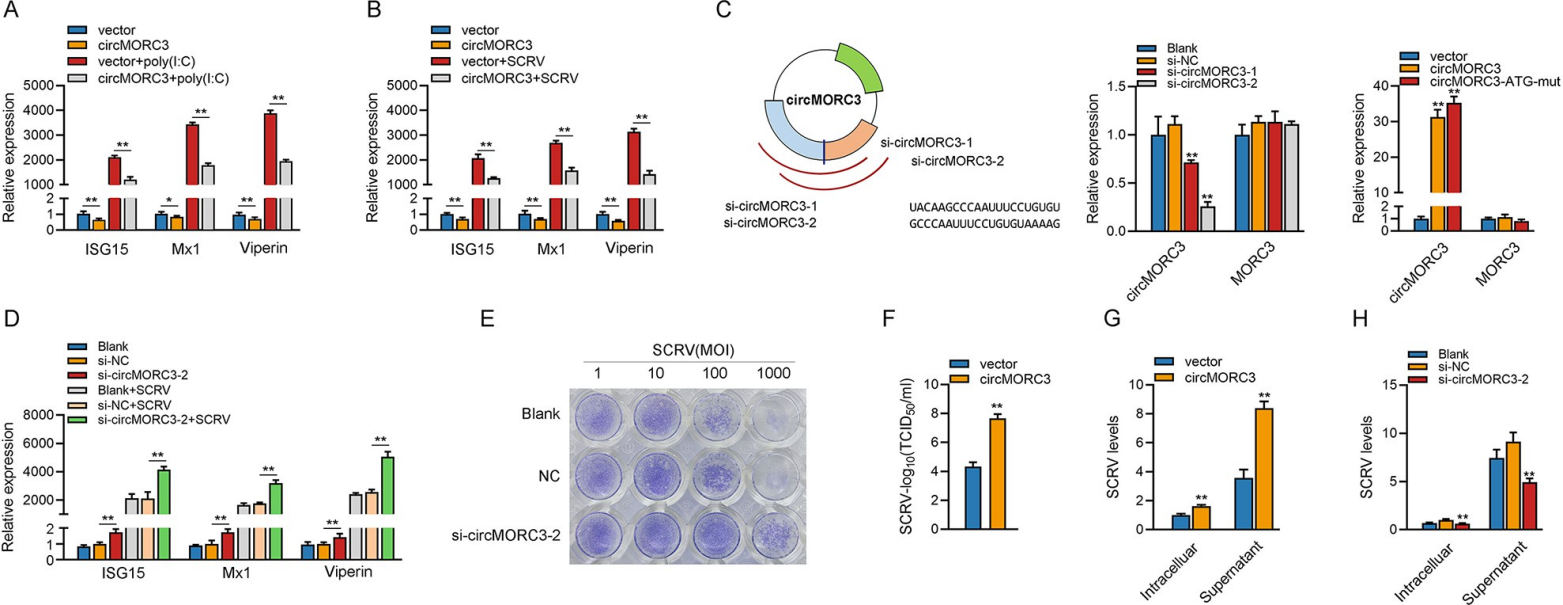
circMORC3 inhibits antiviral innate immunity and favors viral infection. (A and B) qRT-PCR assays were performed to determine the expression levels of antiviral genes in MsbC cells transfected with circMORC3 or control vector (pLC5-ciR) after poly(I:C) stimulation (A) or SCRV infection (B). (C) The schematic diagram of siRNAs. qRT-PCR analysis of circMORC3 and MORC3 mRNA in MsbC cells treated with siRNAs, circMORC3, and circMORC3-ATG-mut. (D) qRT-PCR assays were performed to determine the expression levels of ISG15, Mx1, and Viperin in MsbC cells transfected with si-circMORC3-2 or NC after SCRV stimulation. (E) MsbC cells were fixed with 4% PFA and stained with crystal violet for visualizing CPE. (F) Overexpression of circMORC3 increased virus titers in MsbC cells after SCRV infection, and the viral titer was measured by plaque assay. (G) The qRT-PCR analysis of SCRV mRNA levels in MsbC cells transfected with vector or circMORC3 plasmid after SCRV infection. (H) The qRT-PCR analysis of SCRV mRNA levels in MsbC cells transfected with 100 nM NC or si-circMORC3-2 after SCRV infection. 5.8S rRNA and β-actin were used as the internal control. All data presented as the means ± SE from at least three independent triplicated experiments. **, *p <* 0.01; *, *p <* 0.05 versus the controls.

We further focused on investigating the role of circMORC3 in the innate immune response. Considering that IFN-stimulated genes (ISGs) are important antiviral effectors, we investigated the role of circMORC3 in regulating the expression of ISGs. As shown in Fig 3A, upon poly(I:C) stimulation, circMORC3 could significantly inhibit the expression levels of antiviral genes, such as ISG15, Mx1 and Viperin. Similarly, circMORC3 could significantly negatively regulate the expression levels of these antiviral genes under SCRV infection (Fig 3B). Then, we designed two siRNAs against circMORC3 to explore the biological function of circMORC3 (Fig 3C). Consequently, two siRNAs (si-circMORC3-1 and si-circMORC3-2) decreased the circMORC3 expression level, but such siRNAs did not affect the expression level of linear MORC3 mRNA in MsbC cells. Because si-circMORC3-2 could induce higher inhibitory efficiency, we selected si-circMORC3-2 for the subsequent experiment (Fig 3C). The circMORC3 and circMORC3-ATG-mut could significantly increase the circMORC3 expression levels rather than linear MORC3 mRNA (Fig 3C). As shown in Fig 3D, knockdown of circMORC3 can significantly promote the expression levels of ISG15, Mx1 and Viperin upon SCRV infection. To explore the biological role of circMORC3 in SCRV-induced host cells, we examined the effect of circMORC3 on SCRV replication. After inhibition of circMORC3 expression in MsbC cells, cytopathic effects were attenuated compared with the control upon challenge with different titers of SCRV (Fig 3E). In addition, SCRV replication was also monitored by measuring the SCRV 50% tissue culture infective dose levels in the SCRV-infected MsbC cells. As shown in Fig 3F, circMORC3 increased virus titers in MsbC cells after SCRV infection. As expected, circMORC3 significantly facilitated SCRV replication (Fig 3G), whereas knockdown of circMORC3 significantly suppressed the copy numbers of SCRV genes (Fig 3H). In summary, our finding suggests that circMORC3 negatively regulates the antiviral immune responses of the host.

### 2.4 MORC3-84aa attenuates SCRV induced IFN and ISG expression

**Fig 4:**
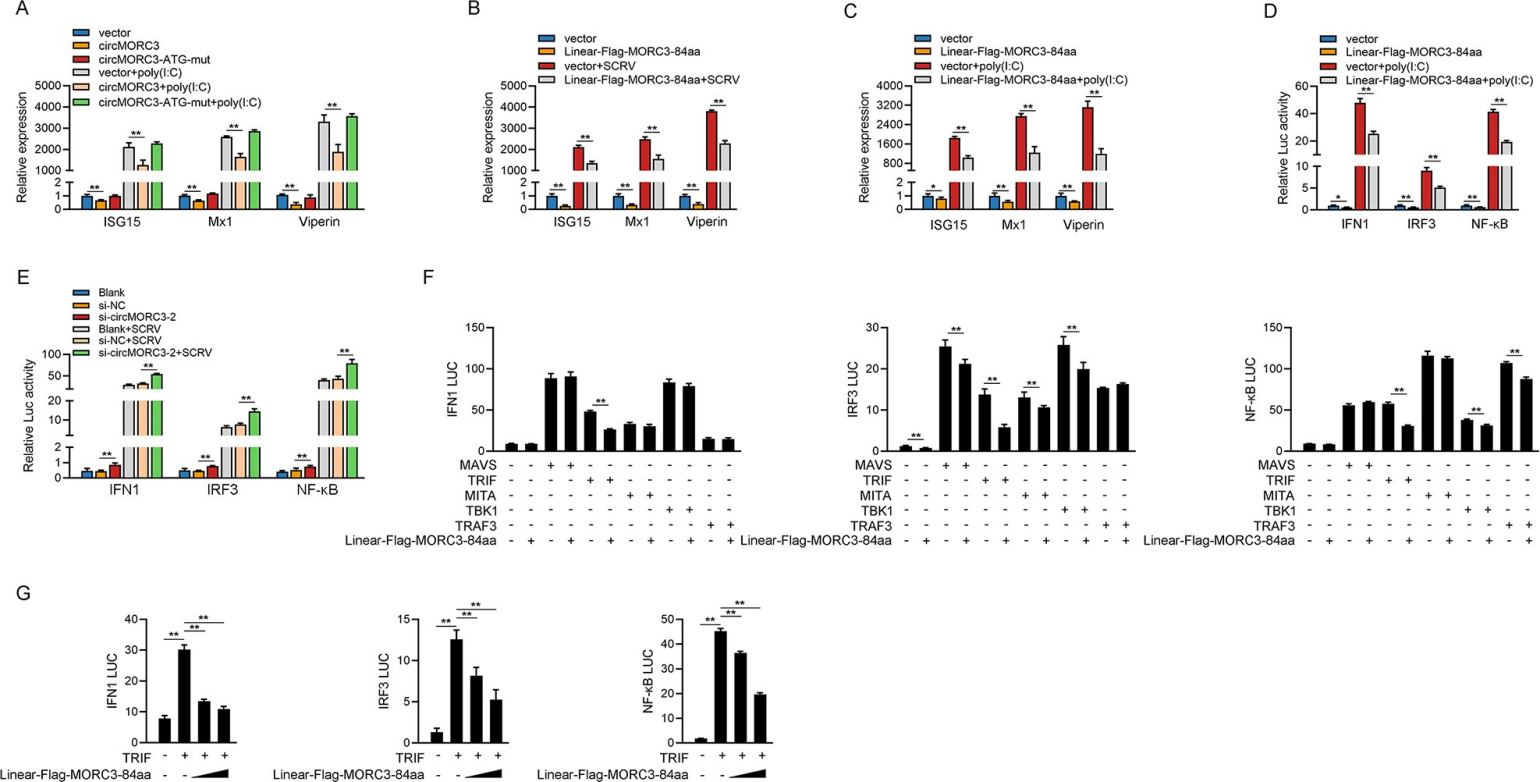
MORC3-84aa attenuates IFN I signaling pathway by targeting TRIF. (A) The qRT-PCR analysis of ISG15, Mx1, and Viperin mRNA in MsbC cells under poly(I:C) treatment. (B) The qRT-PCR analysis of ISG15, Mx1, and Viperin mRNA in MsbC cells with SCRV infection. (C) The qRT-PCR analysis of ISG15, Mx1, and Viperin mRNA in MsbC cells by poly(I:C) stimulation. (D) Relative luciferase activities were detected in MsbC after co-transfection with phRL-TK *Renilla* luciferase plasmid, luciferase reporters, control or Linear-Flag-MORC3-84aa under poly(I:C) stimulation. (E) Relative luciferase activities were detected in MsbC after co-transfection with phRL-TK *Renilla* luciferase plasmid, luciferase reporters, 100 nM NC or si-circMORC3-2 after SCRV infection. (F) Relative luciferase activities were detected in MsbC cells after co-transfected with MAVS, TRIF, MITA, TBK1, and TRAF3 expression plasmids, phRL-TK *Renilla* luciferase plasmid, luciferase reporters, Linear-Flag-MORC3-84aa or pcDNA3.1 plasmids. (G) Relative luciferase activities were detected in MsbC cells after co-transfected with TRIF expression plasmid, phRL-TK *Renilla* luciferase plasmid, luciferase reporters, pcDNA3.1 or concentration gradient of Linear-Flag-MORC3-84aa. All data presented as the means ± SE from at least three independent triplicated experiments. **, *p <* 0.01; *, *p <* 0.05 versus the controls.

As we have proven that circMORC3 was translated into a novel peptide termed MORC3-84aa, we further explored whether circMORC3 or MORC3-84aa contributed to its functions. MsbC cells were stably transfected with the circMORC3, Flag-circMORC3-ATG-mut, and its empty vector. It was found that overexpression of circMORC3 significantly inhibited the expression levels of ISG15, Mx1, and Viperin after poly(I:C) treatment. However, when overexpressing circMORC3 with ATG mutations, there was no significant downregulation of antiviral cytokines (Fig 4A). As shown in Fig 4B, upon SCRV infection, the overexpression of Linear-Flag-MORC3-84aa expression plasmids could significantly inhibit the expression levels of ISG15, Mx1 and Viperin. Consistently, overexpression of Linear-Flag-MORC3-84aa reduced the expression of IFN-stimulated genes (ISG15, Mx1, and Viperin) in MsbC cells after being treated with poly(I:C) (Fig 4C).

Next, we investigated whether MORC3-84aa affected IFN1 activation. As expected, results showed that MORC3-84aa significantly inhibited poly(I:C)-induced activation of IFN1, IRF3 and NF-κB (Fig 4D). As shown in Fig 4E, knockdown of circMORC3 significantly upregulated SCRV-induced activation of IFN1, IRF3 and NF-κB in MsbC cells. To identify the molecular targets of MORC3-84aa in IFN I signaling pathway, the effects of MORC3-84aa on IFN1, IRF3 and NF-κB activation mediated by various adaptors were examined. The results showed that MORC3-84aa inhibited IFN1, IRF3 and NF-κB promoter activation mediated by overexpression of TRIF but not MAVS or MITA (Fig 4F). As shown in Fig 4G, overexpression of MORC3-84aa inhibits the activation of TRIF-induced IFN1, IRF3, and NF-κB in a dose-dependent manner. Altogether, these results indicate that MORC3-84aa suppressed activation of the TRIF-induced IFN I signaling pathway.

### 2.5 MORC3-84aa interacts with TRIF and inhibits its expression

**Fig 5:**
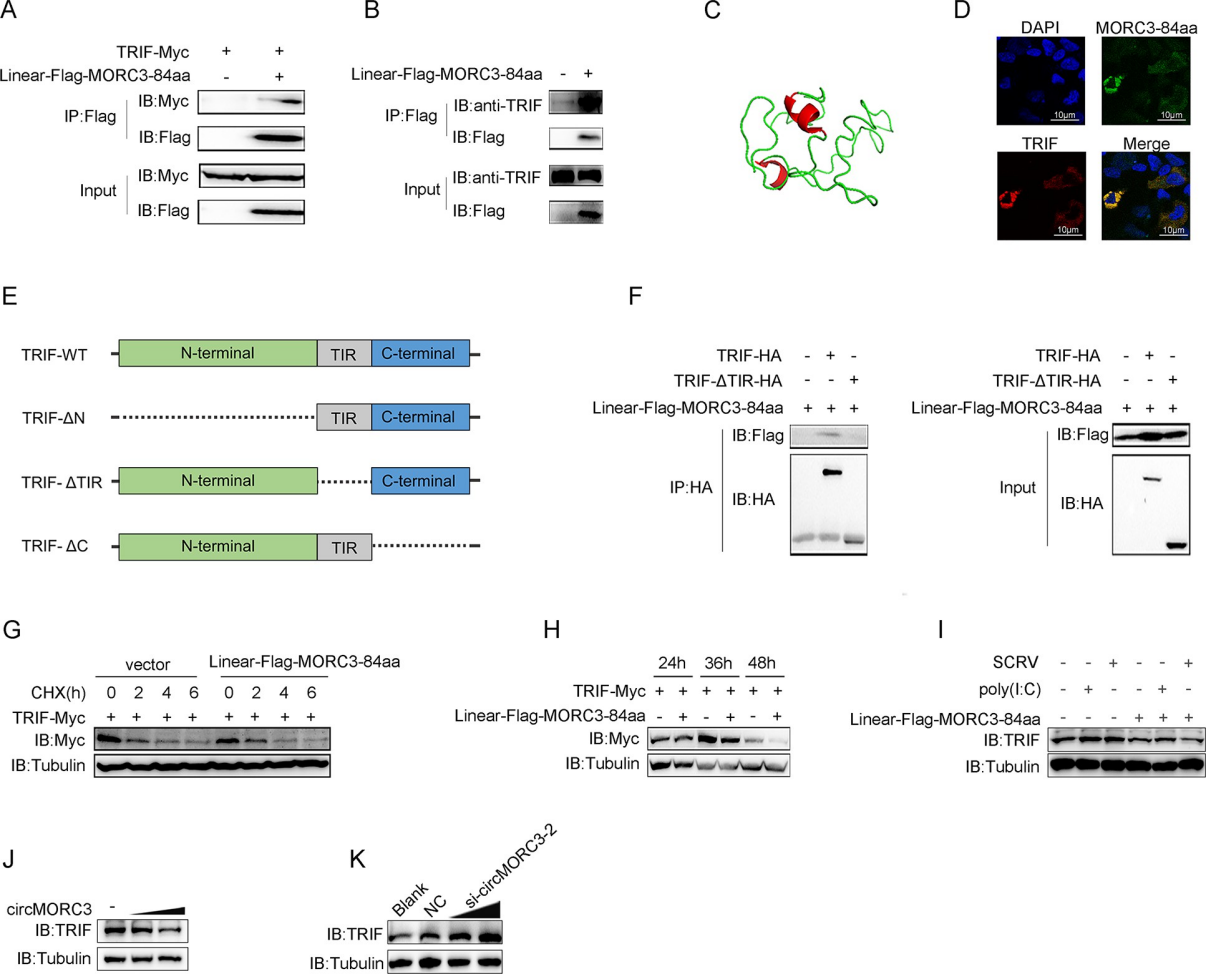
MORC3-84aa directly interacts with TRIF and inhibits its expression. (A) Immunoprecipitation and immunoblot analysis of Linear-Flag-MORC3-84aa and TRIF-Myc in HEK293 cells. (B) Immunoprecipitation and immunoblot analysis of Linear-Flag-MORC3-84aa in MsbC cells. (C) The conformation of MORC3-84aa was predicted by I-TASSER software. (D) Immunofluorescence (IF) was performed in HEK293 cells to determine MORC3-84aa/TRIF colocalization. Scale bar = 10 μm. (E) Schematic illustration shows the truncated mutant of TRIF. (F) IB of whole-cell lysates and proteins immunoprecipitated with Linear-Flag-MORC3-84aa, TRIF, and TRIF mutant from HEK293 cells. (G) EPC cells were transfected with Linear-Flag-MORC3-84aa plasmids and treated with 10 μM CHX. Then, the cell lysates were examined by Western blot. (H) MORC3-84aa inhibited TRIF in a schedule-dependent manner. EPC cells were co-transfected with TRIF-Myc and Linear-Flag-MORC3-84aa and cells were collected at different time points for immunoblot. (I) The TRIF protein level in MsbC cells transfected with either control vector or Linear-Flag-MORC3-84aa under SCRV or poly(I:C) stimulation was detected by Western blot. (J and K) The TRIF protein level in MsbC cells transfected with control vector or circMORC3 (J), and NC or si-circMORC3-2 was detected by Western blot (K). All data presented as the means ± SE from at least three independent triplicated experiments. **, *p <* 0.01; *, *p <* 0.05 versus the controls.

To further confirm the interaction between MORC3-84aa and TRIF, cells were transfected with TRIF-Myc and Linear-Flag-MORC3-84aa expression plasmids. Immunoprecipitation experiments showed that MORC3-84aa was able to interact with TRIF (Fig 5A). Moreover, Flag-tagged MORC3-84aa plasmids were transfected into MsbC cells, and anti-Flag antibody was used to pull down endogenous TRIF. As shown in Fig 5B, immunoprecipitation experiments showed that MORC3-84aa was able to interact with endogenous TRIF. Furthermore, we used I-TASSER software to predict the conformation of MORC3-84aa. MORC3-84aa was visualized in different colors using PyMOL (Fig 5C). In addition, subcellular localization of TRIF and MORC3-84aa was investigated, and immunofluorescence results showed that MORC3-84aa co-localized with TRIF in the cytoplasm (Fig 5D). Based on the mapped structural domains of TRIF, different truncated mutants of TRIF were constructed (Fig 5E). Domain mapping experiments revealed that TRIF interacts with MORC3-84aa through its TIR domain (Fig 5F). Cycloheximide (CHX), a protein synthesis inhibitor, was added to EPC cells transfected with the Linear-Flag-MORC3-84aa vector or pcDNA3.1, and the expression of the TRIF protein was detected at 0, 2, 4, and 6h to further examine the effects of MORC3-84aa on the TRIF protein. As shown in Fig 5G, the overexpression of MORC3-84aa enhanced the degradation speed of TRIF. As expected, MORC3-84aa significantly downregulated TRIF expression in a time-dependent manner (Fig 5H). Subsequently, we examined the effects of MORC3-84aa on endogenous TRIF-mediated signaling. Immunoblot analysis shows that MORC3-84aa significantly inhibits endogenous TRIF regardless of whether the cells were stimulated by poly(I:C) and SCRV (Fig 5I). In addition, circMORC3 overexpression inhibited the expression of endogenous TRIF in a dose-dependent manner in MsbC cells (Fig 5J). To measure MORC3-84aa function in the regulation of endogenous TRIF, we transfected with si-circMORC3-2 to test the levels of endogenous TRIF. As shown in Fig 5K, the downregulation of MORC3-84aa enhanced the protein levels of TRIF in a dose-dependent manner. Taken together, these results suggest that MORC3-84aa can promote the degradation of TRIF protein.

### 2.6 MORC3-84aa targets TRIF for autophagic degradation

**Fig 6:**
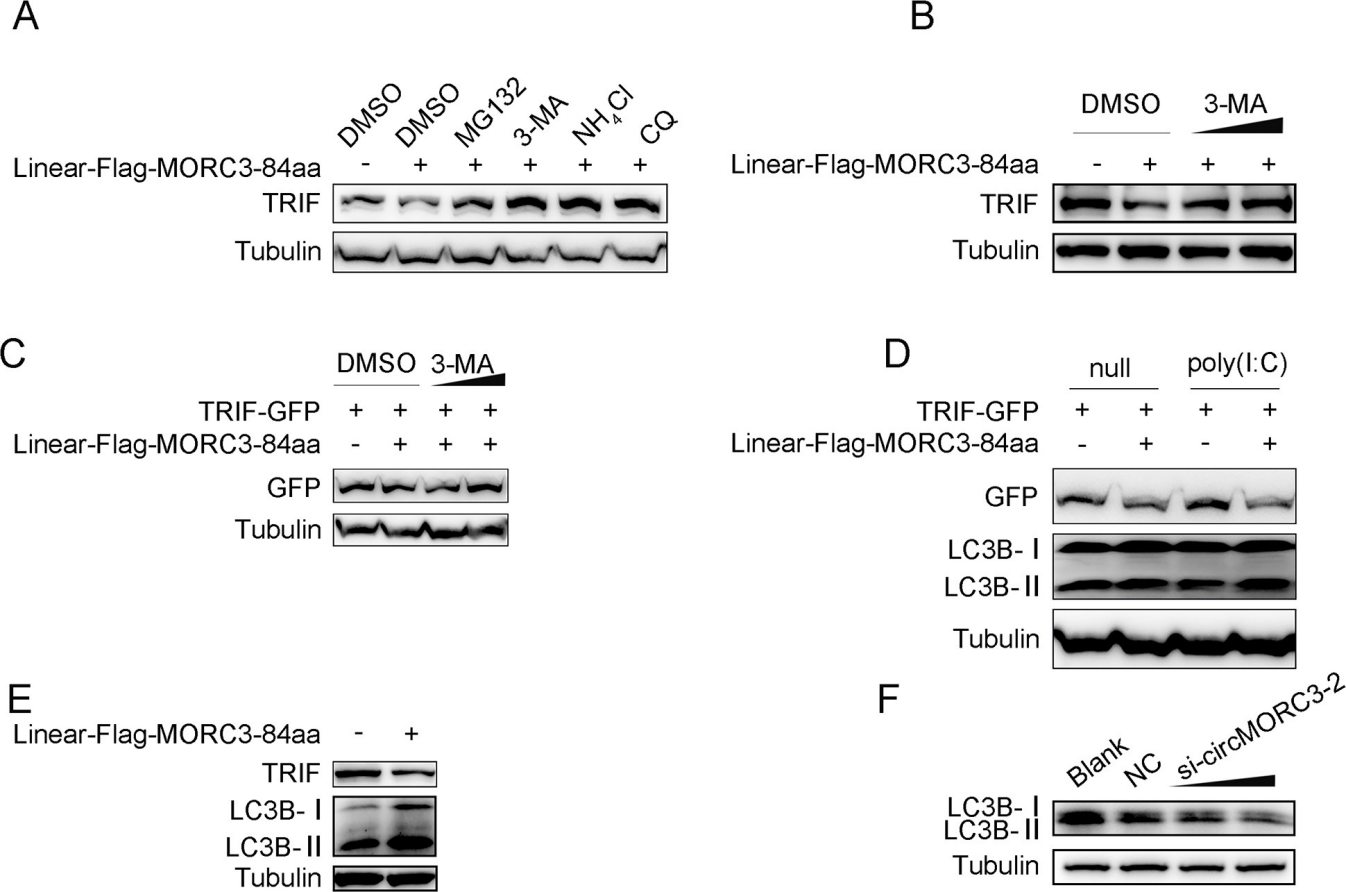
MORC3-84aa promotes autophagic degradation of TRIF. (A) MsbC cells were transfected with Linear-Flag-MORC3-84aa plasmids and treated DMSO, MG132 (15 mM), 3-MA (3 mM), and NH4Cl (20 mM) for 8 h. Then, the cell lysates were examined by Western blot. (B) The TRIF protein level in MsbC cells transfected with control vector or Linear-Flag-MORC3-84aa, followed by DMSO or 3-MA treatment. (C) The TRIF protein level in EPC cells transfected with TRIF-GFP, control vector or Linear-Flag-MORC3-84aa, followed by DMSO or 3-MA treatment. (D) EPC cells were transfected with TRIF-GFP, Linear-Flag-MORC3-84aa or pcDNA3.1. Then, the cell lysates were examined by Western blot. (E) MsbC cells were transfected with Linear-Flag-MORC3-84aa or pcDNA3.1 for 48 h before immunoblot. (F) MsbC cells were transfected with NC or si-circMORC3-2 for 48 h before immunoblot. All experiments were performed in at least three independent experiments.

Next, we examined which pathway was involved in the degradation of TRIF. For this purpose, we used different inhibitors, including proteasome inhibitor MG132, lysosomal inhibitor NH4Cl, autophagy inhibitor 3-methyladenine (3-MA), and chloroquine (CQ), to block the degradation pathway. The results showed that MORC3-84aa-mediated degradation of TRIF was blocked by 3-MA, NH_4_Cl, and CQ, indicating that MORC3-84aa-mediated degradation occurs through autophagic processes (Fig 6A). As shown in Fig 6B, MORC3-84aa-mediated inhibition of endogenous TRIF expression could be reversed by 3-MA in a dose-dependent manner. Furthermore, we transfected GFP-tagged TRIF and Flag-tagged MORC3-84aa or pcDNA3.1 plasmids into EPC cells. The results showed that MORC3-84aa firmly targeted TRIF for degradation, and the addition of 3-MA significantly blocked TRIF degradation in a dose-dependent manner (Fig 6C). MORC3-84aa-mediated degradation of TRIF was enhanced by poly(I:C)-induced activation of autophagy. We found that overexpression of MORC3-84aa caused the increase of LC3B-II after treatment with poly (I:C) (Fig 6D). In addition, we found that when MORC3-84aa mediated endogenous TRIF degradation, the expression of LC3B-II increased (Fig 6E). As shown in Fig 6F, knockdown of MORC3-84aa enhanced the protein levels of LC3B-II in a dose-dependent manner. Collectively, these results suggest that MORC3-84aa likely mediates the degradation of TRIF through autophagy.

### 2.7 MORC3-84aa increases K6-linked ubiquitination of TRIF

**Fig 7:**
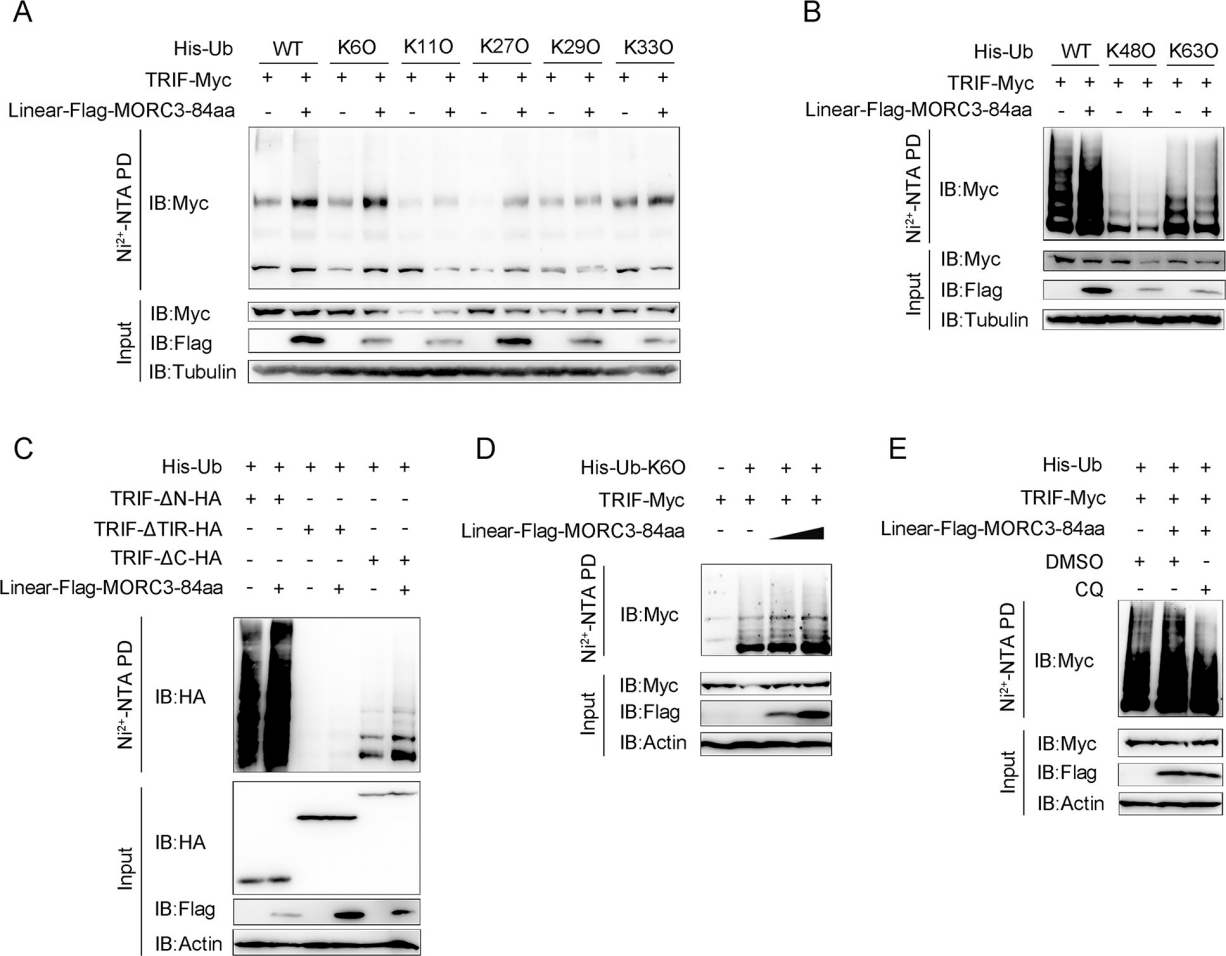
MORC3-84aa promotes K6-linked ubiquitination of TRIF. (A) HEK293 cells were transfected with TRIF-Myc, Linear-Flag-MORC3-84aa, or pcDNA3.1 and His-Ub-WT, His-Ub-K6O, His-Ub-K11O, His-Ub-K27O, His-Ub-K29O or His-Ub-K33O. At 48 h post-transfection, the cells were lysed using guanidinium chloride and purified with Ni^2+^-NTA PD agarose. (B) The ubiquitination of TRIF in HEK293 cells transfected with TRIF-Myc, Linear-Flag-MORC3-84aa, or pcDNA3.1, and His-Ub-WT, His-Ub-K48O or His-Ub-K63O. (C) The ubiquitination of TRIF and its mutants in HEK293 cells transfected with His-Ub-WT, TRIF-HA, mutants of TRIF, Linear-Flag-MORC3-84aa or pcDNA3.1. (D) The K6-linked ubiquitination of TRIF in HEK293 cells transfected with TRIF-Myc, Linear-Flag-MORC3-84aa, or pcDNA3.1 and His-Ub-K6O. (E) Immunoprecipitation analysis of the ubiquitination of TRIF in HEK293 cells transfected with indicated plasmids and treated with CQ for 8 h. All experiments were performed in at least three independent experiments.

Selective autophagy requires the identification of specific goods, which in many cases is achieved through cargo ubiquitination [28]. Then we detected the effects of MORC3-84aa on TRIF ubiquitination. We performed ubiquitination assays using different ubiquitins, including wild-type (WT) ubiquitin, K6O ubiquitin, K11O ubiquitin, K27O ubiquitin, K29O ubiquitin, K33O ubiquitin, K48O ubiquitin, and K63O ubiquitin. The result showed that overexpression of MORC3-84aa promoted ubiquitination of TRIF when WT ubiquitin and K6O ubiquitin were added, but not other ubiquitin (Fig 7A and 7B). Further, we analyzed the effect of MORC3-84aa on ubiquitination in three mutants of TRIF. The result showed that the overexpression of MORC3-84aa did not promote the ubiquitination of TRIF when the TIR domain was absent. However, when the N-terminal and C-terminal domains were absent, TRIF could still be degraded by MORC3-84aa ubiquitination (Fig 7C). As shown in Fig 7D, ubiquitination assays indicated that MORC3-84aa catalyzed K6-linked ubiquitin of TRIF in a dose-dependent manner. Furthermore, when we used CQ to block autophagic degradation, the level of ubiquitin-conjugated TRIF was rescued (Fig 7E), suggesting that the substrates of MORC3-84aa-mediated autophagy are the polyubiquitinated TRIF. Together, these data revealed that K6-linked polyubiquitination of TRIF is required for MORC3-84aa-mediated autophagy.

### 2.8 The host gene MORC3 inhibits antiviral innate immunity

**Fig 8:**
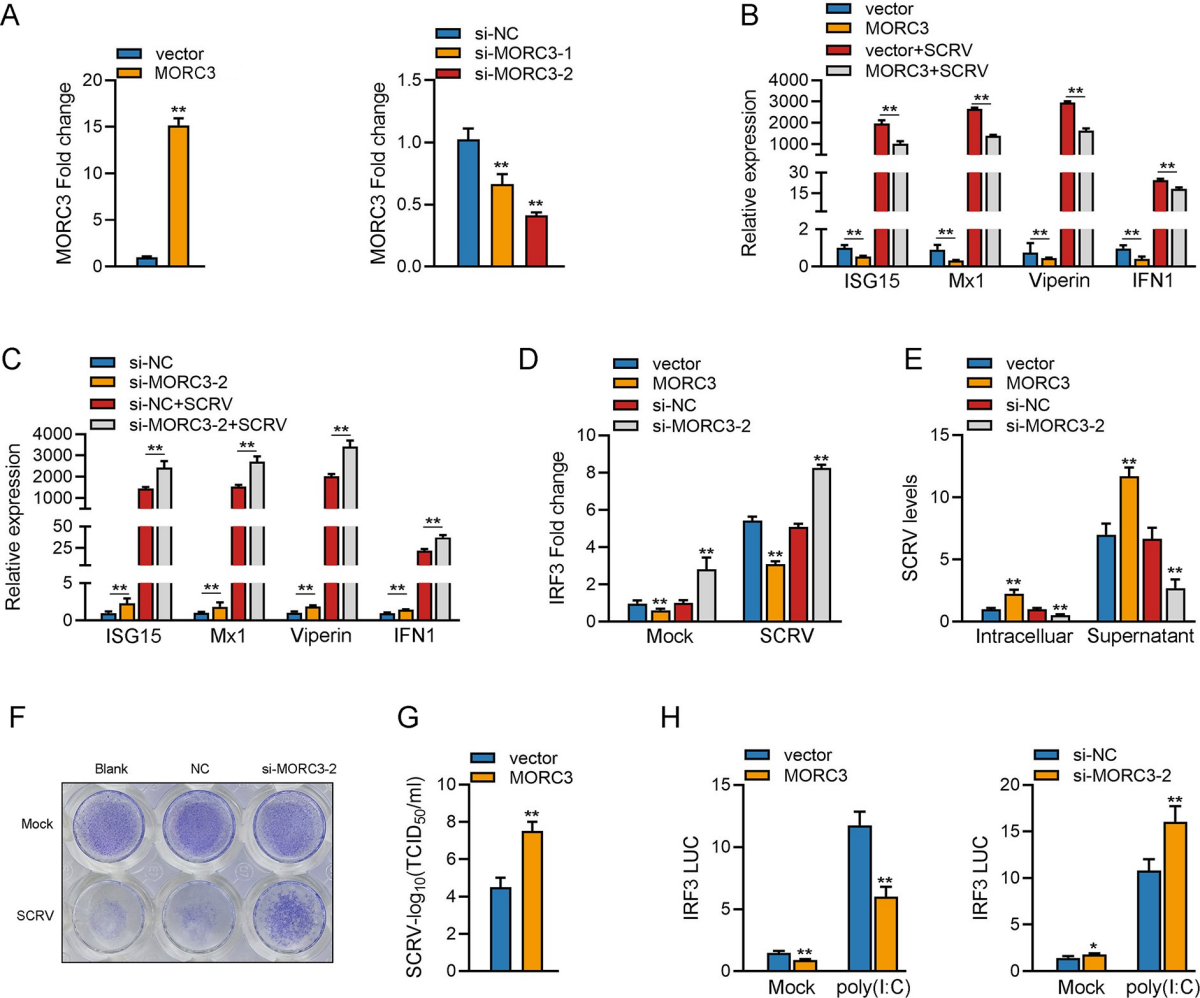
MORC3 inhibits host antiviral innate immunity. (A) qRT-PCR analysis of MORC3 mRNA in MsbC cells transfected with pcDNA3.1, MORC3-Flag plasmids or siRNAs of MORC3. (B) qRT-PCR analysis of ISG15, Mx1, Viperin, IFN1 mRNA in MsbC cells transfected with pcDNA3.1 or MORC3-Flag and followed by SCRV infection. (C) qRT-PCR analysis of ISG15, Mx1, Viperin, and IFN1 mRNA in MsbC cells transfected with NC or si-MORC3-2 under SCRV stimulation. (D) qRT-PCR analysis of IRF3 mRNA in MsbC cells transfected with pcDNA3.1 or MORC3-Flag plasmids and NC or si-MORC3-2 under SCRV infection for 24 h. (E) qRT-PCR analysis of SCRV mRNA levels in MsbC cells transfected with pcDNA3.1 or MORC3-Flag and NC or si-MORC3-2 respectively after SCRV infection. (F) MsbC cells were fixed with 4% PFA and stained with crystal violet for visualizing CPE. (G) Overexpression of MORC3 increased virus titers in MsbC cells after SCRV infection. The culture supernatant was collected from MsbC cells infected with SCRV, and the viral titer was measured by plaque assay. (H) Relative luciferase activities were detected in MsbC cells after co-transfection with IRF3-Luc, phRL-TK, MORC3-Flag or pcDNA3.1 and NC or si-MORC3-2 after poly(I:C) stimulation. All data presented as the means ± SE from at least three independent triplicated experiments. **, *p <* 0.01; *, *p <* 0.05 versus the controls.

To explore the function of MORC3 in the innate immune response, we designed the MORC3 expression plasmids and two MORC3-specific small interfering RNAs. MORC3 mRNA expression was significantly decreased whether silenced by si-MORC3-1 or si-MORC3-2 (Fig 8A). Then, si-MORC3-2 with a better silencing effect on MORC3 was selected for subsequent experiments. As shown in Fig 8B, upon SCRV infection, MORC3 could significantly inhibit the expression levels of ISG15, Mx1, Viperin, and IFN1. However, the results showed that knockdown of MORC3 can significantly enhance the expression levels of ISG15, Mx1, Viperin, and IFN1 upon SCRV infection (Fig 8C). As shown in Fig 8D, knockdown of MORC3 can significantly enhance the expression levels of IRF3 upon SCRV infection while overexpressing MORC3 significantly inhibits the expression levels of IRF3 under SCRV infection. To explore the biological role of MORC3 in SCRV-induced host cells, we examined the effect of MORC3 on SCRV replication. The qRT-PCR results showed that overexpression of MORC3 significantly facilitated SCRV replication, whereas inhibition of MORC3 significantly suppressed SCRV replication (Fig 8E). Knockdown of MORC3 in MsbC cells caused diminished cytopathic effects compared with the control upon challenge with SCRV (Fig 8F). In agreement with this notion, the viral titers in the supernatants of the infected MsbC were confirmed by plaque assay, which showed that the titer in MORC3-overexpressed cells was significantly higher than that of the control cells (Fig 8G). Since IRF3 is an important antiviral transcription factor, we investigated whether MORC3 affects IRF3 activation. The results showed that the overexpression of MORC3 significantly inhibited poly(I:C)-induced activation of IRF3. In addition, the results showed that knockdown of MORC3 can significantly enhance the activation of IRF3 upon poly(I:C) stimulation (Fig 8H). Collectively, these results demonstrated that MORC3 negatively regulates the antiviral responses.

### 2.9 MORC3 regulates IFN induction by targeting IRF3

**Fig 9:**
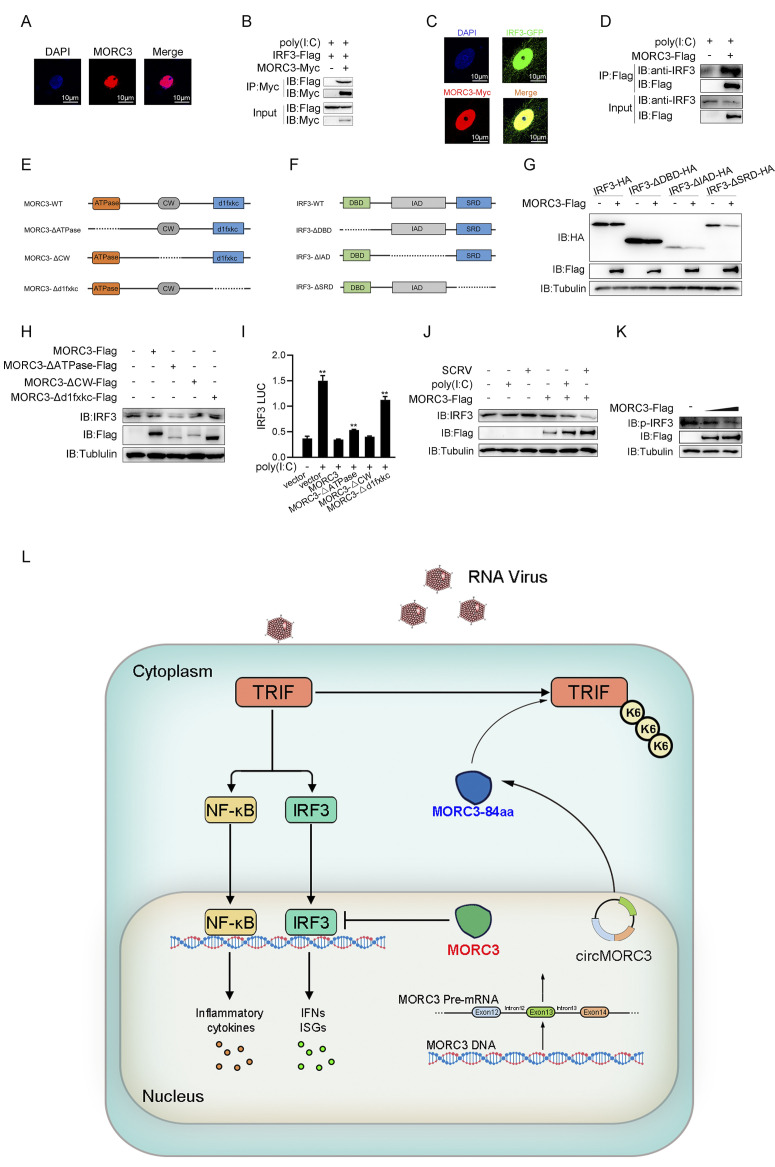
MORC3 promotes degradation of IRF3. (A) The subcellular distribution of MORC3 in EPC cells was examined by immunofluorescence. Scale bars = 10 μm. (B) Immunoprecipitation analysis of the interaction between MORC3 and IRF3 in HEK293 cells transfected with MORC3-Myc and IRF3-Flag plasmids under poly(I:C) treatment. (C) MORC3-Myc were co-transfected with IRF3-Flag into MsbC cells. Immunofluorescence (IF) was performed to determine MORC3/IRF3 colocalization under SCRV infection. Scale bar = 10 μm. (D) Immunoprecipitation and immunoblot analysis of MORC3-Flag in MsbC cells. (E) Schematic diagram of the wild type (WT) and mutants of MORC3. (F) Schematic diagram of the wild type (WT) and mutants of IRF3. (G) Western blot analysis of lysates in HEK293 cells transfected with control vector, MORC3-Flag, IRF3-HA, and three mutants of IRF3. (H) Immunoblot analysis of IRF3 protein level in MsbC cells transfected with MORC3-Flag, three mutants of MORC3 or pcDNA3.1. (I) Effects of MORC3 and its truncations on poly(I:C)-induced IRF3 promoter activation. (J) The IRF3 protein level in MsbC cells transfected with either control vector or MORC3-Flag, followed with SCRV or poly(I:C) stimulation. (K) MsbC cells were transfected with the indicated plasmids for 24 h and then infected with SCRV for 24 h. Immunoblot assays were performed with the indicated antibodies. (L) The proposed working model of circMORC3 in the regulation of antiviral innate immune response. Upon virus infection, the TLR3 sensors undergo conformational change after binding to virus RNA, which promotes the activation of type I IFN expression mediated by TRIF. Furthermore, MORC3 not only directly interacts with IRF3 protein but also suppresses its expression. To evade antiviral immune responses, the expression of circular MORC3 (circMORC3) is induced during viral infection. circMORC3 encodes the MORC3-84aa protein, which enhances the ubiquitination of TRIF at the K6 site. This prompts the activation of the autophagy pathway in TRIF, followed by fusion with lysosomes, ultimately resulting in TRIF protein degradation. As a consequence, the antiviral innate immunity mediated by TRIF is inhibited. All data presented as the means ± SE from at least three independent triplicated experiments. **, *p <* 0.01; *, *p <* 0.05 versus the controls.

To investigate the distribution of MORC3 in cells, we performed immunofluorescence experiments. The result showed that MORC3 mainly localized in the nucleus (Fig 9A). To understand how MORC3 exerts its function in the nucleus, the interaction between MORC3 and IRF3 was analyzed. Immunoprecipitation experiments showed that MORC3 was able to interact with IRF3 under poly (I:C) treatment (Fig 9B). In addition, confocal experiments also showed that MORC3 and IRF3 are located in the nucleus under SCRV infection, suggesting that they may be related (Fig 9C). As shown in Fig 9D, immunoprecipitation experiments showed that MORC3 was able to interact with endogenous IRF3 under poly(I:C) treatment. MORC3 protein has three domains, namely ATPase domain, CW domain, and d1fxkc domain (Fig 9E). We constructed three truncated mutants of IRF3 (ΔDBD, ΔIAD, and ΔSRD) based on their structures (Fig 9F). Apparently, MORC3 efficiently degraded wild-type IRF3 and IRF3 mutated in a single structural domain (Fig 9G). Then, we explored the regulatory effects of MORC3 and the three different MORC3 mutant plasmids on endogenous IRF3. Western blotting experiment results showed that MORC3 suppressed IRF3 expression, and this effect disappeared when the C-terminal domain of MORC3 was deleted (Fig 9H). As expected, MORC3 mutants lack the C-terminal domain (d1fxkc) lost their abilities to inhibit poly(I:C)-triggered IRF3 promoter activation (Fig 9I). Subsequently, we examined the effects of MORC3 on endogenous IRF3-mediated signaling. Immunoblot analysis shows that MORC3 significantly inhibits endogenous IRF3 after poly(I:C) and SCRV stimulation (Fig 9J). Functionally, MORC3 inhibits the activation of the phosphorylation of IRF3 (Fig 9K). These data indicate that MORC3 suppressed IFN activation by targeting IRF3.

Based on the data we obtained, we proposed a working model for circMORC3 and MORC3 (Fig 9L). When the innate immune system suffers viral invasion, TLR3 initiates a potent anti-viral immune response by binding to double-stranded RNA ligands and recruits TRIF, inducing the production of antiviral cytokine [29,30]. To evade immunity, MORC3 interacts directly with IRF3 and inhibits IFN induction by targeting IRF3. In the subsequent second phase, circMORC3 induced by SCRV enhanced K6-linked ubiquitination of TRIF by encoding the novel peptide MORC3-84aa. Subsequently, MORC3-84aa induced autophagy and transported TRIF into the autophagosome to fuse with lysosomes for TRIF degradation, contributing to the termination of TRIF-mediated antiviral immune responses.

## 3. Discussion

In recent years, circRNAs have received increasing attention due to their important roles in disease occurrence, development, diagnosis, and treatment [31]. As proteomics and translation technology have grown in popularity and increased in precision and accuracy, it was discovered that circRNAs are translatable [32]. Moreover, it is recognized that long ncRNAs (lncRNAs) and circular RNAs (circRNAs) contain sORFs that can be translated into functional small peptides [33, 34]. In the present study, we report that circMORC3 is self-cyclized by exon12-exon14 and intron12-intron13 from MORC3. The preliminary experimental results showed that the expression of circMORC3 was significantly enhanced after infection with SCRV. Then, we identified that the circMORC3 was translatable and encoded a novel isoform, MORC3-84aa. Previous studies have shown that translation of circRNAs can proceed by a cap-independent mechanism that requires internal ribosome entry site (IRES) or N6-methyladenosine (m6A) modifying sequences [35]. For example, circβ-catenin translates through IRES-mediated translation into a novel 370 aa protein [36]. We found that circMORC3 is translated in a cap-independent manner using a cis-regulatory element termed IRES. The sequence of circMORC3 was assessed using IRESfinder to determine whether it contains an IRES structure [37], and the 174nt sequence was identified. Then, we experimentally demonstrated the translation initiation capacity of IRES in circMORC3.

Previous studies have proved the key role of these circRNAs and their protein products in diseases, including hepatocellular carcinoma, gastric cancer, and multiple myeloma malignancy [38–40]. In addition, the host genes of circRNAs may be involved in the mechanism of circRNAs in disease development. For example, circBUB1B_544aa and the host gene BUB1B act synergistically to exacerbate multiple myeloma [40], circFOXO3 negatively regulates the host gene FOXO3 in osteoarthritis through activation of autophagy [16]. However, the relationship between protein-coding circRNAs and host genes in innate immunity has not been well studied. In the present study, the expression of MORC3 and circMORC3 during SCRV infection is divided into two phases. In the first phase, host gene MORC3 expression was upregulated, but circMORC3 expression did not change significantly. In the second stage, the expression of host gene MORC3 returned to normal levels, whereas the expression of circMORC3 was significantly upregulated. The expression trends of circMORC3 and host gene implied a close relationship with the viral infection process.

Since our finding demonstrated that circMORC3 was significantly upregulated in viral infections and encodes MORC3-84aa, we further evaluated whether MORC3-84aa was responsible for the antiviral response of teleost fish. We found that MORC3-84aa inhibits the induction of antiviral cytokines by mediating the degradation of TRIF. In addition, MORC3-84aa inhibits IRF3 and NF-κB signaling pathways by targeting TRIF. Previous studies have shown that TRIF is an essential adaptor protein for TLRs-mediated innate immune response [29,41]. When the associated TLRs are stimulated by their ligands, it leads to recruitment of TRIF molecules and activation of IRF3. In addition, IRF3 forms a dimer and is translocated into the nucleus. As a result, IRF3 induces the expression of type I IFN genes [42]. Some negative regulatory mechanisms of TRIF have been reported in immune cells. For example, CD11b functions as a negative regulator of TLR-triggered inflammatory responses by directly inhibiting the MyD88 and TRIF pathways [43]. Other proteins involved in this process are TRIM38 and E3 ligase WWP2 [44, 45]. TRIF consists of an N-terminal region, a TIR structural domain and a C-terminal region. The TIR structural domain of TRIF is essential for binding the TIR structural domain of TLR3 [46]. In this study, our results showed that MORC3-84aa was significantly associated with the full-length and TIR structural domains of TRIF, while TRIF lacking the TIR structural domain lost the ability to interact with MORC3-84aa, suggesting that MORC3-84aa may block TRIF binding to downstream linker proteins by binding to the TIR structural domain. In addition, we found that MORC3-84aa not only inhibits IRF3 and NF-κB signaling pathways by mediating TRIF but also inhibits the induction of antiviral cytokines by mediating the degradation of TRIF.

Autophagy is a conserved degradation pathway and accumulating evidence has revealed the crosstalk between autophagy and immune responses. For example, loss of the autophagy-related gene Atg16l1 promoted the accumulation of TRIF and downstream signaling in macrophages [47]. Previous studies have shown that ubiquitination-mediated autophagy plays a major role in preserving innate immune homeostasis. For example, NEDD4 catalyzes the K27-linked poly-ubiquitination of TBK1 at K344, which serves as a recognition signal for cargo receptor NDP52-mediated selective autophagic degradation [48]. Afterwards, the PB1 protein of influenza A virus preferentially associated with a selective autophagic receptor neighbor of BRCA1 (NBR1) that recognizes K27-linked polyubiquitination of MAVS and delivers it to autophagosomes for degradation [49]. In our study, MORC3-84aa markedly enhanced the K6-linked ubiquitination of TRIF. Then MORC3-84aa enhanced the delivery of ubiquitinated TRIF to autophagosome for autophagic degradation, which contributes to the immune suppression of type I IFN signaling.

MORC3 (NXP2/KIAA0136/ZCWCC3) was first discovered through a sequencing project aimed at understanding its structure. In addition, MORC3 is a member of the MORC gene family and the functional region mapping of MORC3 reveals that MORC3 is a nuclear matrix protein that contains a putative RNA binding site in its nuclear matrix binding domain contains a putative RNA binding site, which is essential for transcriptional regulation [50,51]. Previous studies have shown that there are two or three coiled-coil structural domains at the C-terminus. Moreover, the coiled-coil structural domain is an important structure that regulates protein-protein interactions [52]. Here, we found that MORC3 can inhibit IFN induction and the phosphorylation level of IRF3. When the MORC3 protein lacks the C-terminal structural domain, it loses the ability to repress poly(I:C)-triggered activation of the IRF3 promoter. Therefore, the C-terminal domain might be an essential structure of MORC3 in regulating IRF3. Previous studies have shown that IRF3 (interferon regulatory factor 3) serves as an indispensable transcription factor for type I IFN production. After receiving the upstream signal of TRIF, IRF3 goes through diverse processes to activate the type I IFN signaling, including phosphorylation, dimerization, translocation to the nucleus, and transcriptional activity of type I IFN genes [53]. During the early stages of viral infection, it is possible that the host gene MORC3 interacts with nuclear IRF3 to potentially facilitate the degradation of phosphorylated IRF3. This interaction may play a role in regulating the host immune response. In the subsequent stages of viral infection, there is a possibility that MORC3-84aa, a protein encoded by the circMORC3, interacts with TRIF in the cytoplasm and may contribute to the degradation of TRIF protein. Consequently, our results indicate circMORC3 and host gene MORC3 can synergistically inhibit TRIF and IRF3 in the TLR signaling pathway at different stages of viral infection, promoting viral immune escape.

In conclusion, we demonstrate that MORC3-84aa and MORC3 synergistically inhibit the TLR signaling pathway at different stages of viral infection. circMORC3 encoded MORC3-84aa peptide significantly enhances autophagic degradation of TRIF by promoting K6-linked ubiquitination. Furthermore, we found that MORC3 not only inhibits IRF3 protein expression but also inhibits the phosphorylation level of IRF3. In addition, our study expands the understanding of the function of circRNAs and host genes in viral immune escape.

## 4. Materials and methods

### 4.1 Ethics statement

All animal experimental procedures were performed in accordance with the National Institutes of Health’s Guide for the Care and Use of Laboratory Animals, and the experimental protocols were approved by the Research Ethics Committee of Shanghai Ocean University (No. SHOU-DW-2018-047).

### 4.2 Sample and challenge

*M*. *miiuy* (~50 g) was obtained from Zhoushan Fisheries Research Institute, Zhejiang Province, China. Fish were acclimated in aerated seawater tanks at 25°C for six weeks before experiments. *Siniperca chuatsi rhabdovirus* (SCRV) infection was performed as described [9]. Briefly, fish were challenged with 200 μl SCRV at a multiplicity of infection (MOI) of 5 through intraperitoneal injection. As a comparison, the control group was challenged with 200ul of physiological saline. Subsequently, fish were sacrificed at different time points, and the tissues were collected for RNA extraction.

### 4.3 Cell culture and treatment

*M*. *miiuy* kidney cells (MKC) and *M*. *miiuy* swim bladder cells (MsbC) were cultured in L-15 medium (HyClone) containing 15% fetal bovine serume (Gibco), 100 μg/ml streptomycin, and 100 U/ml penicillin, and placed in a 26°C incubator [9]. Fish EPC cells (Epithelioma papulosum cyprini cells) were cultured at 26°C, EPC cells were cultured in medium 199 (HyClone) supplemented with 10% fetal bovine serum, 100 U/mL penicillin and 100 mg/mL streptomycin. HEK293 cells were cultured in a humidified incubator at 37°C. HEK293 cells were cultured in DMEM (HyClone) supplemented with 10% FBS, 100 U/mL penicillin, and 100 mg/mL streptomycin [54]. For stimulation experiments, MsbC cells were stimulated with SCRV, poly(I:C), and LPS at different time, and the cells were used for qRT-PCR analysis and immunoblot analysis [9,55]. For virus stimulation experiments, MsbC cells were challenged with SCRV at a multiplicity of infection (MOI) of 5 and harvested at different times for RNA extraction.

### 4.4 Plasmids construction

The CDS of TRIF, TBK1, IRF3, and MORC3 were amplified from the *M*. *miiuy* cDNA through standard PCR methods, and then the expression plasmids were cloned into pcDNA3.1 vector (Invitrogen) with Myc, HA, and Flag tag, respectively. The fusion plasmid of TRIF-GFP was cloned into GFP vector. TRIF mutations, including TRIF-ΔN (dN), TRIF-ΔTIR (dTIR), and TRIF-ΔC (dC), were generated by PCR using specific primers based on the TRIF-HA recombinant plasmid. MORC3 mutations, including MORC3-ΔATPase, TRIF-ΔCW, and TRIF-Δd1fxkc, were generated by PCR using specific primers based on the MORC3-Flag recombinant plasmid. To construct circMORC3 overexpression vector, the full-length circMORC3 cDNA was amplified by specific primer pairs and cloned into pLC5-ciR vector (Geneseed Biotech). The His-tagged ubiquitin-WT (His-Ub) was purchased from Addgene. His-Ub-K6O, His-Ub-K11O, His-Ub-K27O, His-Ub-K29O, His-Ub-K33O, His-Ub-K48O and His-Ub-K63O were expression plasmids for His-tagged Lys-6-, Lys-11-, Lys-27-, Lys-29-, Lys-33-, Lys-48- and Lys-63-only ubiquitin mutants. All recombinant plasmids were affirmed by DNA sequencing. The primer sequences are listed in S1 Table.

### 4.5 RNA interference

MORC3- and circMORC3-targeted small interfering RNAs were designed by the online website. RNA interference sequences for circMORC3 are the following: si-circMORC3-1, 5’-UACAAGCCCAAUUUCCUGUGU-3’; si-circMORC3-2, 5’-GCCCAAUUUCCUGUGUAAAAG-3’. RNA interference sequences for MORC3 are the following: si-MORC3-1, 5’-CAGAAAGUAGAUGAAUUGAUU-3’; si-MORC3-2, 5’-GGAUCCUACUCACAACAAACA-3’. The scrambled control RNA sequence was 5’ -UUCUCCGAACGUGUCACGUTT- 3’. The siRNAs and a nontargeting control siRNA (NC) were purchased from Gene Pharma (Shanghai, China). MsbC were transfected with 100 nM of each siRNA using the Lipofectamine RNAiMAX (Invitrogen).

### 4.6 Luciferase reporter assay

Luciferase reporter plasmids IFN1, NF-κB, and IRF3 were characterized previously [54,56]. EPC cells were seeded in 48-well plates and transfected with plasmids containing full length or deleted circMORC3 IRES. After 48 h, luciferase activities were determined by the Dual-Luciferase Reporter Assay Kit (Promega). To determine the functional regulation of MORC3-84aa, cells were co-transfected TRIF overexpression plasmid or MORC3-84aa overexpression plasmid, together with NF-κB, IFN1, IRF3 luciferase reporter gene plasmids and phRL-TK plasmid. At 48 h post-transfection, the cells were lysed for reporter activity using the dual-luciferase reporter assay system (Promega). si-circMORC3-2 or NC were transfected with NF-κB, IRF3, IFN1 luciferase reporter gene plasmids and phRL-TK plasmid. At 48h posttransfection, the cells were lysed for reporter activity. All the luciferase activity values were achieved against the *Renilla* luciferase control. Transfection of each construct was performed in triplicate in each assay.

### 4.7 RNA extraction and quantitative real-time PCR

Total RNA was extracted using the TRIzol Reagent (Invitrogen) and the cDNA was synthesized using the FastQuant RT Kit (Tiangen) which contains DNase treatment of RNA to eliminate genomic contamination, following the manufacturer’s instructions. The expression profiles of each gene were conducted by using the SYBR Premix Ex Taq Kit (Vazyme), as previously described [54]. The quantitative real-time PCR was conducted in an Applied Biosystems QuantStudio 3 (Thermo Fisher Scientific). β-actin was used as internal controls for mRNA respectively. Primer sequences are listed in S1 Table. The procedure is as follows: 95°C for 3 min, 40 cycles of 95°C for 10s, 60°C for 10s, and 95°C for 15s.

### 4.8 Immunoblot assay

The cells were washed three times using sterile and cold PBS. Then, cells were lysed by NP-40 lysis buffer (0.5 M Tris (pH 7.4), 0.5 M EDTA, 150 mM NaCl, 1% NP-40) containing protease inhibitor cocktail (Bitake) and PMSF (Beyotime). Protein concentrations were measured via BCA assay (Pierce) and equalized with extraction reagent. Equal amounts of extracts were mixed with 2X SDS loading buffer, loaded onto SDS-PAGE, and then transferred onto nitrocellulose membrane (Merck Millipore) using semi-dry blotting system (Bio-Rad Trans Blot Turbo System). The membranes were washed thrice using TBST buffer (20 mM Tris, 150 mM NaCl, 0.1% Tween 20, pH 7.5) and blocked at room temperature using 5% dried skimmed milk by TBST diluted on rocker platform for 90 min. The membranes were then incubated with primary antibodies at 4°C overnight. Primary antibodies used in this study were against Myc, HA, Flag, and Tubulin (Beyotime). The membranes were washed thrice using TBST and incubated with secondary antibody at room temperature on the rocker platform for 60 min. Finally, immunoreactive proteins were detected with Western Bright ECL, and digital imaging was performed by cold CCD camera (Tanon).

### 4.9 RNase R treatment

The RNAs (10 μg) from MKC and MsbC cells were treated with RNase R (3 U/μg, Epicenter) and incubated for 30 min at 37°C. Then, the treated RNAs were reverse transcribed with divergent primer or convergent primer and detected by qRT-PCR and RT-PCR assay followed by nucleic acid electrophoresis.

### 4.10 Nuclear and cytoplasmic extraction

According to the manufacturer’s instructions, cytoplasmic and nuclear RNA were isolated and purified from MKC and MsbC cells using a cytoplasmic and nuclear RNA purification kit (Norgen Biotek).

### 4.11 Nucleic acid electrophoresis

The cDNA and gDNA PCR products were investigated using 2% agarose gel electrophoresis with Tris-acetate-EDTA (TAE) running buffer. DNA was separated by electrophoresis at 100 V for 30 min. The DNA marker was Super DNA marker (100 to 10,000 bp) (CWBIO). The bands were examined by UV irradiation.

### 4.12 Co-immunoprecipitation

Coimmunoprecipitation (Co-IP) experiments, the cells were cultured into 6 cm^2^ plate overnight and co-transfected 4 μg total plasmids. After 24 h from transfection, the cells were washed thrice with cold PBS. The cells were lysed with western and IP lysis buffer containing protease inhibitor cocktail and PMSF at 4°C for 20 min on a rocker platform. The cellular fragment was separated by centrifugation at 12000 g for 10 min at 4°C. After centrifugation, the supernatant was transferred into a new centrifuge tube and incubated with protein A+G (Santa cruz) and monoclonal anti-Myc or anti-Flag (Abbkine) overnight at 4°C with soft agitation. The following day, IP protein was collected by centrifugation at 2500 g for 5 min at 4°C. Then, the beads were washed three times with western and IP lysis buffer and resuspended in 60 μl SDS loading buffer. Immunoprecipitates and whole cell lysates were analyzed by immunoblotting.

### 4.13 Ubiquitination assay

For analysis of the ubiquitination of TRIF in cells, cells were transfected with plasmids expressing TRIF-Myc, His-ubiquitin (WT) and Linear-Flag-MORC3-84aa in HEK293 cells. Ubiquitination assays with His-ubiquitin or His-ubiquitin-mutants were performed by affinity purification on Ni^2+^-NTA Resin (Denaturation type) as described previously [57].

### 4.14 Immunofluorescence

Cells grown on glass coverslips in 24-well plates were fixed with 4% paraformaldehyde for 15 min after cells were washed with PBS for three times. Then cells were permeabilized with 0.5% TritonX-100 in PBS (PBST) for 15 min. After blocking with 5% BSA for 60 min, the cells were incubated with primary antibody at 4°C for 10 h. Cells were washed three times with PBS and incubated with fluorescent-dye conjugated secondary antibody for 60 min at room temperature. After counterstaining with DAPI staining for 10 min, Fluorescence signals of cells were acquired using a fluorescence microscope (Leica).

### 4.15 Plaque assay

The cells were washed three times using sterile PBS and were fixed with 4% paraformaldehyde (PFA). After removing PFA, the cells were washed three times with sterile ddH_2_O. Then add crystal violet staining solution to the cells and stain for 30 min at room temperature. Wash with sterile ddH_2_O three times and dry at room temperature.

### 4.16 Statistical analysis

Data are expressed as the mean ± SE from at least three independent triplicated experiments. Student’s t-test was used to evaluate the data. The relative gene expression data was acquired using the 2 ^-ΔΔCT^ method [58], and comparisons between groups were analyzed by one-way analysis of variance (ANOVA) followed by Duncan’s multiple comparison tests. A value of *p* < 0.05 was considered significant.

## Supporting information

S1 Data Data that underlies Figure panels 1A, 1B, 1F, 1G, 2B, 3A, 3B, 3C, 3D, 3F, 3G, 3H, 4A, 4B, 4C, 4D, 4E, 4F, 4G, 8A, 8B, 8C, 8D, 8E, 8G, 8H, 9I. (XLSX)

S1 Table PCR primer information in this study. (DOCX)

S1 Fig Detection of circMORC3. (A) The relative copy numbers of circMORC3 and MORC3 in liver samples after SCRV infection were detected by absolute quantitative PCR, actin is control gene. (B) The relative copy numbers of circMORC3 and linear MORC3 in MsbC cells measured by absolute quantitative PCR after SCRV infection, actin is control gene. (C) Computational analysis was used to identify the stem-loop like structure of precursor in circMORC3.
(PDF)

S2 Fig Verification of circMORC3 overexpression plasmid. (A) Sanger sequencing validation of circMORC3, Flag-circMORC3, and Flag-circMORC3-ATG-mut overexpression plasmid capable of producing circular circMORC3. (B) qRT-PCR detection of the efficiency of circMORC3, Flag-circMORC3, and Flag-circMORC3-ATG-mut overexpression plasmids in producing circMORC3.
(PDF)
