# Supplementary material for: circMORC3-encoded novel protein negatively regulates antiviral immunity through synergizing with host gene MORC3
Source: PLoS Pathog. 2023 Dec 27;19(12):e1011894. doi: 10.1371/journal.ppat.1011894 (PMC10775979; doi:10.1371/journal.ppat.1011894)
Supplement: S1 Table — (DOCX) [file ppat.1011894.s002.docx]

**S1** **Table** PCR primer information in this study.

| **Primers** | **Sequences (5’-3’)** |
| --- | --- |
| **Vector construction** | |
| circMORC3-F | CGGAATTCTAATACTTTCAGTGTAAAAGAGGAGCACAAAGCC |
| circMORC3-R | CGGGATCCAGTTGTTCTTACCAGGAAATTGGGCTTGTAGCT |
| Flag-circMORC3-F | TACAAAGACGATGACGACAAGTAACGAAACATCGGAGTTTGATTT |
| Flag-circMORC3-R | GTCGTCATCGTCTTTGTAGTCGATGTGCGGCGGAGGCTC |
| Linear-Flag-MORC3-84aa-1F | GACGATGACGACAAGAAGCTTGCCACCATGCAGATCATCATACGTGGCG |
| Linear-Flag-MORC3-84aa-1R | GTGCTCCTCTTTTACACAGGAAATTGGGCTTGTAGCTG |
| Linear-Flag-MORC3-84aa-2F | CCTGTGTAAAAGAGGAGCACAAAGCCA |
| Linear-Flag-MORC3-84aa-2R | TGATGGATATCTGCAGAATTCTTAGATGTGCGGCGGAGGCTC |
| Flag-circMORC3-ATG-mut-F | GCCTCGGAAACAGATCATCATACGTGGCGTCA |
| Flag-circMORC3-ATG-mut-R | TGATCTGTTTCCGAGGCTTCAGATAAAGAATACTG |
| Linear-Flag-circMORC3-F | TTTCCTGGTGGATCCTAGCTAACAACTCCATACTTT |
| Linear-Flag-circMORC3-R | CTAGGATCCACCAGGAAATTGGGCTTGTAGCTG |
| IRES-WT-F | CTATCGATAGGTACCGAGCTCTGTAAAAGAGGAGCACAAAGCCA |
| IRES-WT-R | CAGTACCGGAATGCCAAGCTTAACTCCGATGTTTCGTTAGATGTG |
| IRES-DEL-1-F | CTATCGATAGGTACCGAGCTCTGTAAAAGAGGAGCACAAAGCCA |
| IRES-DEL-1-R | CAGTACCGGAATGCCAAGCTTTCGATCTCGAGGAGCAGTTCTT |
| IRES-DEL-2-F | CTATCGATAGGTACCGAGCTCCGCCATCAGTTCCTCCAACA |
| IRES-DEL-2-R | CAGTACCGGAATGCCAAGCTTAACTCCGATGTTTCGTTAGATGTG |
| MORC3-HindⅢ-F | GACGATGACGACAAGAAGCTTATGGCGGCGCAAACGGAC |
| MORC3-EcoRI-R | TGATGGATATCTGCAGAATTCTTATTTATTTATTGCTCTGTTCTTCACTACA |
| MORC3-ΔHATPase-F | ATTCCACCAGCCACAAAGCCAGCCTGCAGGACA |
| MORC3-ΔHATPase-R | CTTTGTGGCTGGTGGAATTTGTATGAAGAAACT |
| MORC3-ΔCW-F | CCAGATCAAGTGGACTCTGATGATGAGATGCGA |
| MORC3-ΔCW-R | AGAGTCCACTTGATCTGGTCGCTTCATGGCGTC |
| MORC3-Δd1fxkc-F | GGAAAGCGACAAAACCACTGATTCATCTGCACAA |
| MORC3-Δd1fxkc-R | GTGGTTTTGTCGCTTTCCTGAGCTGTAGCCTGC |
| TRIF-F | GACGATGACGACAAGAAGCTTATGAGCCGCGAGGGAGAA |
| TRIF-R | TGATGGATATCTGCAGAATTCCTAAAGACATTGCTCATCTGAATCATC |
| TRIF-dN-F | AGAGACGTCGCATTTGTCATCTTTCACGCGCCA |
| TRIF-dN-R | GACAAATGCGACGTCTCTTAGTCCCGTCCCCTG |
| TRIF-dC-F | GAGAGCAAGTCAGATGAGCAATGTCTTTAGGAATT |
| TRIF-dC-R | CTCATCTGACTTGCTCTCATCGAGTGGAACTAG |
| TRIF-dTIR-F | GAGGCGCAATTTTACAGCTTTGAGAAAAAAATAAGAGCATC |
| TRIF-dTIR-R | GCTGTAAAATTGCGCCTCTTCCTTCTCCTCTTC |
| IRF3-KpnI-F | CGGGGTACCATGTCTCATTCTAAACCTCTGCTCATC |
| IRF3-XbaI-R | TGCTCTAGAGTGTCAGTACAGCTCCATCATCTC |
| IRF3-dDBD-EcoRI-F | CCGGAATTCAACTCTAGTGCTGGATCC |
| IRF3-dDBD-EcoRI-R | CCGGAATTCAGCGTAATCTGGAACATCGT |
| IRF3-dIAD-EcoRI-F | CCGGAATTCCCAGACAACAGGCCTTGGGAG |
| IRF3-dIAD-EcoRI-R | CCGGAATTCATCTCCATCTCTGGTCTTGTT |
| IRF3-dSRD-EcoRI-F | CCGGAATTCCTCGAAGAGATGATGGAGCTG |
| IRF3-dSRD-EcoRI-R | CCGGAATTCGGCGCCGCCTCCAACAGCCA |
| **Real-time PCR** | |
| circMORC3-divergent-qRT-F | TACAAGCCCAATTTCCTGTGT |
| circMORC3-divergent-qRT-R | GTTGGAGGAACTGATGGCGTC |
| circMORC3-convergent-qRT-F | CCGCTATCCTGGTGTATC |
| circMORC3-convergent-qRT-R | CAGCAACTGTGGGAACTA |
| IL-1β-qRT-F | CATAAGGATGGGGACAACGAG |
| IL-1β-qRT-R | TAGGGGACGGACACAAGGGTA |
| IL-8-RT-F | AGCAGCAGAGTCTTCGT |
| IL-8-RT-R | TCTTCGCAGTGGGAGTT |
| GAPDH-qRT-F | GAGCACTGTCCACGCCATCA |
| GAPDH-qRT-R | CCGTTCAGCTCAGGGACGA |
| U6-qRT-F | TGCGAGTAGCAGACCA |
| U6-qRT-R | CACGAGACCGAAACAC |
| IFN1-qRT-F | TACGATGGCTAATAACTCC |
| IFN1-qRT-R | CATTGACAAAGTGCTCCA |
| Mx1-qRT-F | GCTGCTTGTTTACTCCCA |
| Mx1-qRT-R | ACCTGCATCATCTCCCTC |
| ISG15-qRT-F | TGAACGGACAGAAGACGC |
| ISG15-qRT-R | TGAGGAATACCTGCATGG |
| Viperin-qRT-F | ACCCGTCCAAGTCCATAC |
| Viperin-qRT-R | TCATGTCAGCTTTGCTCC |
| SCRV-qRT-F | GGGCTGGATGATAGACGATTG |
| SCRV-qRT-R | TGGCGGAGGTGCTTGATATGG |
| 5.8S rRNA-qRT-F | AACTCTTAGCGGTGGATCA |
| 5.8S rRNA-qRT-R | GTTTTTTTTTTTTTTTGCCGAGTG |
| β-actin-qRT-F | GAGCCGCACGCTTCTTT |
| β-actin-qRT-R | CTGCTGTAGCCGAGGAC |
| IRF3-qRT-F | TCCGCTTAGTCTACAGCCCT |
| IRF3-qRT-R | CCAGGATGCATTGGGTCAGT |
