# Supplementary figures and images for: circMORC3-encoded novel protein negatively regulates antiviral immunity through synergizing with host gene MORC3
Source: PLoS Pathog. 2023 Dec 27;19(12):e1011894. doi: 10.1371/journal.ppat.1011894 (PMC10775979; doi:10.1371/journal.ppat.1011894)

A

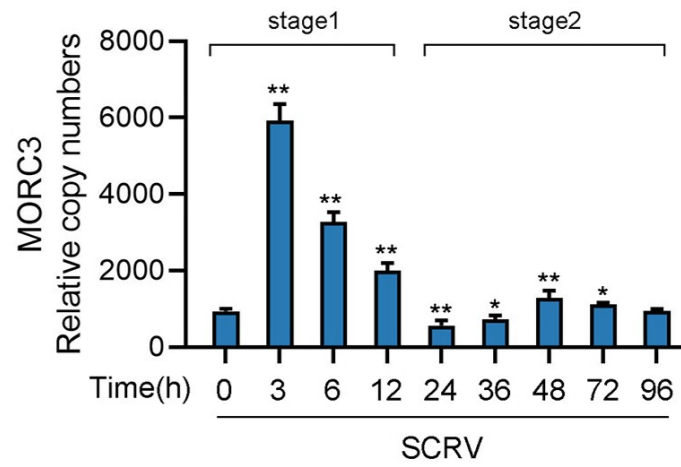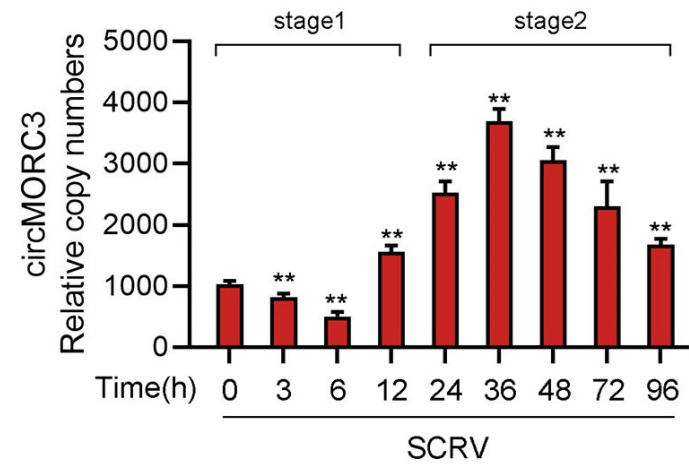

B

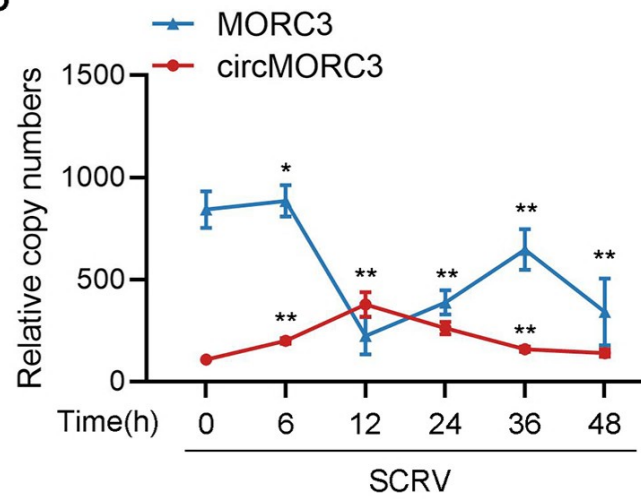

C

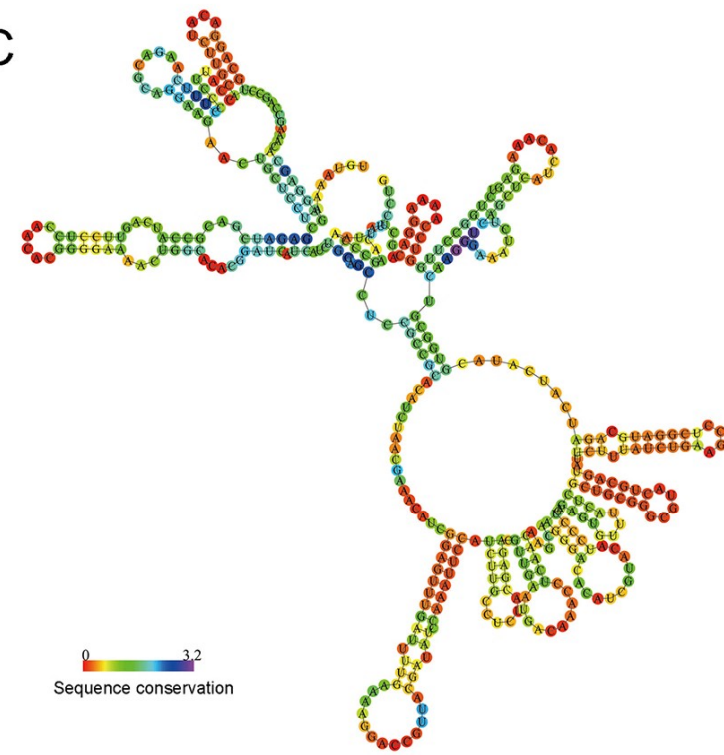

Supplement: S1 Fig — (A) The relative copy numbers of circMORC3 and MORC3 in liver samples after SCRV infection were detected by absolute quantitative PCR, actin is control gene. (B) The relative copy numbers of circMORC3 and linear MORC3 in MsbC cells measured by absolute quantitative PCR after SCRV infection, actin is control gene. (C) Computational analysis was used to identify the stem-loop like structure of precursor in circMORC3. (PDF) [file ppat.1011894.s003.pdf]

A

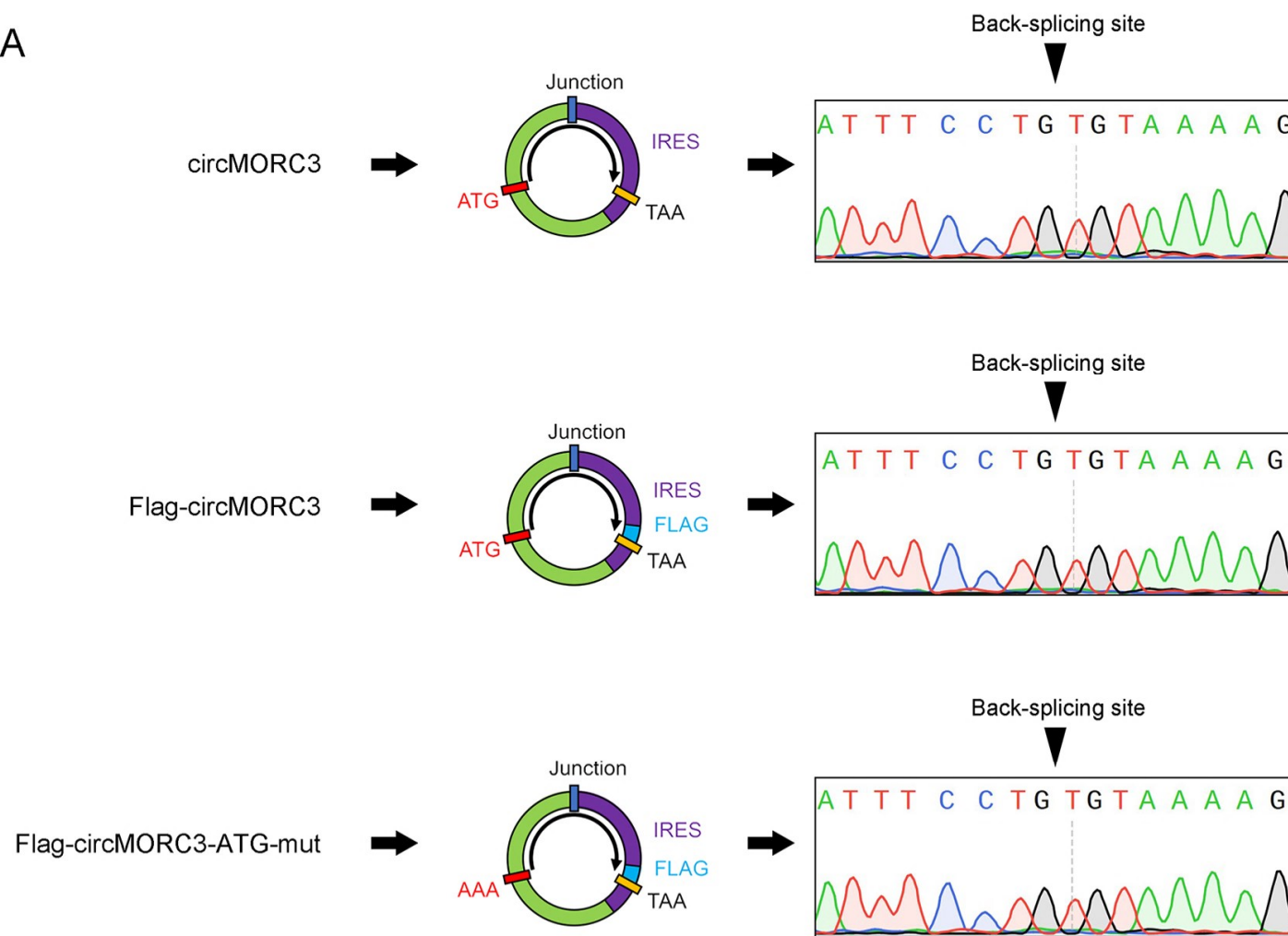

B

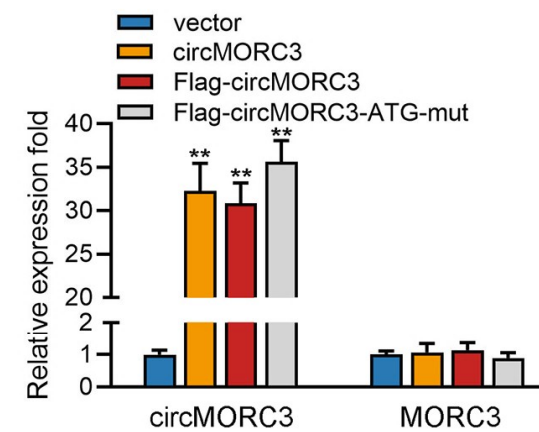

Supplement: S2 Fig — (A) Sanger sequencing validation of circMORC3, Flag-circMORC3, and Flag-circMORC3-ATG-mut overexpression plasmid capable of producing circular circMORC3. (B) qRT-PCR detection of the efficiency of circMORC3, Flag-circMORC3, and Flag-circMORC3-ATG-mut overexpression plasmids in producing circMORC3. (PDF) [file ppat.1011894.s004.pdf]
